# FBXL16: a new regulator of neuroinflammation and cognition in Alzheimer’s disease through the ubiquitination-dependent degradation of amyloid precursor protein

**DOI:** 10.1186/s40364-024-00691-w

**Published:** 2024-11-21

**Authors:** Liqun Qu, Yong Tang, Jianhui Wu, Xiaoyun Yun, Hang Hong Lo, Linlin Song, Xingxia Wang, Huimiao Wang, Ruilong Zhang, Menghan Liu, Cairen Wang, Jerome P. L. Ng, Xianjun Fu, Io Nam Wong, Vincent Kam Wai Wong, Betty Yuen Kwan Law

**Affiliations:** 1 Dr. Neher’s Biophysics Laboratory for Innovative Drug Discovery, State Key Laboratory of Quality Research in Chinese Medicine, Macau University of Science and Technology, Macau S.A.R, Avenida Wai Long, Macau, 999078 China; 2 Sichuan Key Medical Laboratory of New Drug Discovery and Druggability Evaluation, Luzhou Key Laboratory of Activity Screening and Druggability Evaluation for Chinese Materia Medica of Southwest Medical University, Luzhou, 646000 China; 3 Research Institute for Marine Traditional Chinese Medicine, Key Laboratory of Marine Traditional Chinese Medicine in Shandong Universities, Shandong Engineering and Technology Research Center on Omics of Traditional Chinese Medicine, Shandong University of Traditional Chinese Medicine, Jinan, 250355 China; 4 Qingdao Key Laboratory of Research in Marine Traditional Chinese Medicine, Qingdao Academy of Chinese Medical Sciences Shandong University of Traditional Chinese Medicine, Qingdao Key Technology Innovation Center of Marine Traditional Chinese Medicine’s Deep Development and Industrialization, Qingdao, 266114 China; 5 Faculty of Medicine, Macau University of Science and Technology, Macau, 999078 China

**Keywords:** FBXL16, Ubiquitin, Alzheimer’s disease, Neuroprotection, Amyloid precursor protein

## Abstract

**Background:**

Activating the ubiquitin-proteasome system to dismantle disease- related proteins such as tau, β-amyloid, APP, and α-synuclein is an important focus in the research of neurodegenerative proteinopathy. By analyzing the serum RNA extracted from wild-type and Alzheimer’s disease (AD) transgenic mice at different ages (4, 8, and 12 months), this study revealed a new protective role of FBXL16 in AD, primarily through facilitating the degradation of disease-related proteins via the ubiquitin proteasome system.

**Methods:**

Proteomic analysis were conducted using protein lysates from HEK293 cells overexpressing FBXL16 to identify potential interacting proteins that interact with FBXL16. Subsequent experiments demonstrated that FBXL16 promotes the proteasomal degradation of the APP protein, as evidenced by co-immunoprecipitation with MG132 and cycloheximide (CHX), immunohistochemistry (IHC) and immunocytochemistry (ICC). Memory and cognitive improvements were observed in 3×Tg AD mice through the use of a lentivirus-mediated approach to generate a brain-specific AD mouse model overexpressing FBXL16 via stereotaxic injection. Furthermore, a brain-specific conditional knockout (cko) FBXL16 mouse model was generated and employed to further confirm the functional role of FBXL 16 in AD via various behavioral tests including Morris water maze and Y-maze.

**Results:**

The level of FBXL16 in the brains of transgenic APP/PSEN mice with AD decreased with age. Accelerated degradation of APP was observed when FBXL16 was overexpressed in the hippocampi of these AD mice via a lentivirus. This process led to notable improvements in cognitive impairments and reductions in neuroinflammation. Further studies using proteomics and bioinformatics techniques identified transcription factors and binding proteins associated with FBXL16, providing deeper insights into the potential role of FBXL16 in the regulation of AD. Finally, the in vivo effects of FBXL16 deficiency were further substantiated in cko mice, which overexpress Aβ but specifically lack FBXL16 in the brain region.

**Conclusions:**

These findings suggest that FBXL16 could be a new regulator of AD. These findings provide a foundation for further research into drug development and potential therapeutic strategies to combat other related neurodegenerative proteinopathies.

**Supplementary Information:**

The online version contains supplementary material available at 10.1186/s40364-024-00691-w.

## Background

Alzheimer’s disease (AD) is an age-related neurodegenerative disorder characterized by progressive cognitive impairment. With the aging of the population worldwide, the incidence of neurodegenerative diseases is gradually increasing, and these diseases have become a heavy burden and medical problem for families and society [1, 2]. The 2021 WHO Global Status Report states that there are now 40–50 million people with AD, and this figure is expected to exceed 100 million by 2050 [3]. Although the exact etiology and pharmacological target of AD remain unclear, studies have reported various AD risk factors, including genetic defects, the β-amyloid (Aβ) cascade, free radical damage, abnormal calcium metabolism, an imbalanced cholinergic system, neuroinflammation and aluminum toxicity. At present, available pharmacological treatments, including donepezil, rivastigmine, galantamine, and memantine, are prescribed to improve symptoms via the modulation of neurotransmitter levels without alleviating the pathogenesis of AD [4]. Most candidate compounds for the treatment of AD are developed by targeting Aβ, tau, or inflammation, and small molecule compounds and monoclonal antibodies targeting amyloidogenic amyloid precursor protein (APP) processing and Aβ deposition are still the main candidates for the development of AD drugs. For example, monoclonal antibodies, including aducanumab and lecanemab, have been recently approved by the FDA for the treatment of AD by targeting Aβ [5].

The ubiquitin-proteasome system (UPS) pathway is essential for preventing intracellular protein accumulation [6], especially in the central nervous system, through the degradation and removal of mutant and misfolded proteins such as Tau, APP, and α-synuclein to prevent neurological diseases [7]. β-Amyloid precursor protein originates from the endoplasmic reticulum (ER) and matures in the Golgi apparatus before being delivered to the cell membrane. The amyloid cleavage of APP was linked to the deposition of amyloid-β in the brains of AD patients [8]. Recent studies have shown that damage to the UPS pathway can lead to the aggregation of disease proteins and neurodegenerative diseases such as AD. Ubiquitin mutants can be detected in the brains of AD patients whose proteasome activity is inhibited [9]. Under normal conditions, Aβ is quickly degraded without causing any harm. However, Aβ can accumulate and become pathogenic due to the abnormal function of the ubiquitin–proteasome pathway in AD patients [10]. In addition to Aβ, ubiquitinated proteins, including neurofibrillary tangles (NFTs), have also been found in the brains of AD patients [11]. This evidence suggested that improving the abnormal UPS pathway could be beneficial for AD. F-box and leucine-rich repeat protein 16 (FBXL16), an E3 ubiquitin ligase, is a rarely studied F-box protein that contains an N-terminal pro-rich domain and a C-terminal LRR domain in addition to its core common conserved F-box motif [12]. The C-terminal region contains secondary structures that specifically recognize and bind to substrates that in turn participate in the ubiquitin-mediated protein degradation pathway. Deficiencies in the FBXL16 gene lead to perinatal lethality in mice [13], and depletion of FBXL16 promotes the differentiation of mouse embryonic stem cells [14], suggesting that FBXL16 plays an essential role in physiological and developmental processes. Despite the central role of the FBXL protein in the management of various cellular processes, such as protein degradation, cell cycle progression, cell proliferation, apoptosis, migration, invasion, and metastasis [15], little is known about the molecular and physiological roles of FBXL16 in the ubiquitous degradation of the pathogenic proteins such as APP.

In this study, with the preliminary observation that the level of FBXL16 in transgenic APP/PSEN mice decreased in an age-dependent manner, the molecular mechanism of FBXL16 in both in vivo and in vitro AD models was first investigated. Given the unreported role of FBXL16 in the ubiquitination of APP in both lentiviral FBXL16-overexpressing and FBXL16-conditional knockout (cko) mice, proteomics and bioinformatics analyses were adopted to evaluate the neuroprotective role of FBXL16 in the degradation of APP, cognitive ability and neuroinflammation in AD mouse models, which has provided scientific evidence for the future development of AD therapy.

## Materials and methods

### Cell lines and reagents

The HEK293 and H4 cell lines were purchased from Sichuan Aiki Biotechnology Co. SHSY-5Y cell lines were purchased from ATCC (ATCC No: CRL-2266). APP/PSEN mice (APPSwePSEN1dE9, stock numbers, #N000175) were purchased from Nanjing Institute of Biomedical Research, Nanjing University. 3×Tg AD mice (APPSwe, tauP301L, stock numbers, #004807) were purchased from the Jackson Laboratory. Flag-hFBXL16 and pLV-hFBXL16-HA were purchased from VectorBuilder, Inc. HA-ubiquitin and pEGFP-N1-APP were purchased from Addgene. TaqDNA polymerase was purchased from Takara; the reverse transcription system (T4 DNA ligase; restriction endonucleases BamHI, XhoI, HindIII, EcoR1, and Xba1) was purchased from Fermentas; *Escherichia coli* DH5a was purchased from Tiangen Biochemical Technology Co., Ltd.; aminoglutamicin, kanamycin, 10% SDS, 1·5MTris-HCL PH8.8, 1 M Tris-HCL (pH 6·8), 30% polypropionamide, Tris and glycine were purchased from Solarbio; DNA markers were purchased from Tiangen Chemical Company; fetal bovine serum, OPTI-MEM medium, DMEM, NaCl, chloroform, isopropanol, anhydrous hexanol, EDTA, and NP-40 were purchased from Sinopharm Chemical Reagent Company; Flag-CMV2F empty control vector, cell lysis buffer, BSA, Tween-20, Triton X-100, APS, TEMED and PMSF were purchased from Sigma; pCMV-N-HA and pCMV-N-Myc empty control vectors were purchased from Beyotime; and the protein quantification BCA kit and cell nuclear extraction kit were purchased from Thermo, USA. The PVDF membrane was purchased from Millipore. The AXYGEN gel recovery kit and AXYGEN plasmid small extraction kit were purchased from AXYGEN. Real-time fluorescence quantitative SYBR was purchased from Beijing All-Style Gold Company. The FBXL16-, Flag- and APP-specific antibodies were purchased from Thermo. HA, Myc, and ubiquitin antibodies were purchased from Cell Signaling Technology. Horseradish peroxidase secondary antibodies, protein G agarose, ubiquitin (WT), actin, and HA were purchased from Santa Cruz.

### Protein identification by mass spectrometry

For protein identification by mass spectrometry, 0 to 2 µg of the Flag-FBXL16 plasmid was transfected into HEK293 cells. The FBXL16 protein was precipitated and eluted from the magnetic beads by using Flag magnetic beads (Beyotime) and glycine (0·1 M, pH = 2·8) and then neutralized with 0·5 M Tris-HCl. The identity and quantity of the eluted FBXL16 were verified by Western blotting. The samples were subsequently sent to Shenzhen Chengqi Biotechnology Co., Ltd. The proteins were digested into peptide mixtures via trypsin and ionized via MALDI, after which the peptide ions at specific mass‒to-nucleus ratios were separated via a mass analyzer. Protein identification was performed by comparing of the actual spectra with the theoretical primary and secondary mass spectra peaks generated by protease digestion of proteins.

### Behavioral tests

#### Morris water maze

A circular pool with a diameter of 120 cm and a height of 50 cm was prepared with a water depth of 30 cm at a water temperature of 22 °C. A white platform with a diameter of 9 cm was placed 1 cm below the water surface in the center of the first quadrant, and a camera above the center of the pool was used to record the trajectory and escape latency of the mice. The mice were first subjected to a 5-day localization and navigation experiment. Briefly, the mice were gently placed in different quadrants of the pool for each experiment and allowed to explore freely for 90 s. If the hidden platform was not found within 90 s, the mice were guided to rest on the platform for 30 s. The experiment was conducted once a day, each time starting from a different quadrant of the pool and with the trajectory was recorded. After 5 days of navigation, the platform in the quadrant was removed, and the pool environment was left unchanged. Then, the mice were allowed to explore freely for 60 s, and their trajectories were recorded. The time spent in the first quadrant and the number of times the mice crossed the original platform position were also recorded to evaluate the spatial orientation and memory ability of the mice. The mice were kept as quiet and mildly illuminated as possible, without special odors, to avoid fear and distraction.

#### Y maze

The Y maze consisted of 3 equal arms (50 cm × 18 cm × 35 cm) with an angle of 120 degrees between each pair of arms. In this experiment, chow was placed on the two upper arms, and the mice were placed at the bottom of the Y-maze (the starting arm), while the trajectory of the mice was recorded for 10 min. The following indicators were recorded: (i) total number of entries: the number of times the mice entered each arm of the maze (the criterion was that the mouse entered each arm once with all four feet); and (ii) the number of alternations: the number of times the mice entered all three arms of the Y-maze in succession.

### Western blotting

After treatment, the cells were lysed in RIPA lysis buffer (50 mM Tris-HCl (pH 7·4), 150 mM NaCl, 1% Triton X-100, 1% sodium deoxycholate, 0·1% SDS) supplemented with protease and phosphatase inhibitors for protein extraction. Protein lysates were collected and then quantified via the Bio-Rad DCTM Protein Assay Kit (Bio‐Rad, Hercules, CA, USA). Appropriate amounts of protein were denatured at 95–100 °C for 5 min. Equal amounts (µg) of total protein lysate were loaded into each well of a 10% SDS‒polyacrylamide gel (SDS-PAGE) immersed in Tris‒glycine Tris-glycine SDS running buffer (25 mM Tris, pH 8·3; 192 mM glycine; 0·1% SDS) at 120 V. The separated proteins were subsequently transferred to a PVDF membrane in Tris‒glycine Tris-glycine transfer buffer (25 mM Tris, pH 8·3 192 mM glycine, 20% (v/v) methanol). The membranes were blocked with 5% skim milk in TBST for 1 h at room temperature. The corresponding primary antibodies were added to the membrane, which was incubated overnight at 4 °C. After the membrane was washed with TBST, the secondary antibody was added, and the membrane was incubated at room temperature for 1 h. Actin was used as a control for normalization of the band intensity. The chemiluminescence signal was detected by using an AI800 system (GE Healthcare).

### Immunostaining

HEK293 cells were seeded at a density of 1 × 10^5^ cells/well in a 6-well plate. After APP was cotransfected with various concentrations of Fbxl16 via Lipofectamine 3000, the cells were fixed with 4% paraformaldehyde (Sigma) at room temperature for 15 min. The fixed cells were then permeabilized with PBS containing 1% Triton X-100 (Sigma‒Aldrich). After fixation, the cells were incubated overnight with a mixture of FBXL16 and APP antibodies at 4 °C, followed by incubation with Alexa Fluor 488-labeled goat anti-rabbit IgG (H + L) and Alexa Fluor 555-labeled donkey anti-mouse IgG (H + L) for 1 h at room temperature in the dark. Nuclei were stained with 1 µg/ml 4′,6-diamidino-2-phenylindole (DAPI) (Sigma). The sample was mounted and sealed with FluorSave™ Reagent on a microscope slide where a drop of Antifade Mounting Medium (Millipore) was placed. Images were captured via a confocal laser microscope (Leica).

### Coimmunoprecipitation (Co-IP)

HEK293 cells were grown in culture flasks, and when the density reached 80-90% confluence, Flag-FBXL16 and Myc-APP vectors were transfected into HEK293 cells, which were incubated at 37 °C for 24 h in a 5% CO_2_ incubator. The cells were then washed and lysed with RIPA lysis buffer on ice for 10 min with intermittent vortex shaking. Protein lysates were then collected by centrifugation at 12,000×g for 15 min at 4 °C. The supernatant containing the proteins was collected and quantified using a Bradford protein assay kit (Bio-Rad). Equal concentrations and volumes of the protein were added to SDS loading buffer and boiled at 95–100 °C for 5 min. The remaining protein lysate was divided equally into 2 portions and then incubated with anti-Flag or anti-Myc antibodies overnight at 4 °C on a shaker. After incubation, the mixture was placed on a magnetic stand for 10 s for separation, 500 µl of 1×TBS was added, and the mixture was resuspended in anti-Myc magnetic beads by gentle pipetting. The beads were subsequently washed three times. Then, 100 µl of 1× SDS‒PAGE loading buffer was added to every 50 µl of the original bead mixture, which was subsequently heated at 95 °C for 5 min. Finally, the tube was placed on a magnetic stand for 10 s to separate and remove the supernatant for SDS‒PAGE electrophoresis or Western blot detection.

### Subcellular localization

A sterile coverslip was placed in a 35 mm cell culture dish, and 2 × 10^4^ PC12, Neuro-2 A or SHSY-5Y cell suspensions were added and incubated in the dish overnight. The next day, the cells were washed with PBS, fixed with 4% PFA at room temperature for 15 min and then incubated with 5% BSA at room temperature for 2 h. The cells were incubated overnight at 4°C with 1 mL of primary antibodies against FBXL16 (diluted 1:250) and APP (diluted 1:250). Then, 1:250 dilutions of mixed fluorescent secondary antibodies, including Alexa Fluor 488-labeled goat anti-rabbit and Alexa Fluor 555-labeled donkey anti-mouse, were added to the cells, which were incubated at 37 °C for 1 h. After washing with PBST, diluted DAPI (1:1000) was added to the cells, which were incubated for 10 min at room temperature in the dark. The coverslips with cells were then removed, air-dried, sealed, and observed via confocal microscopy.

### Chromatin immunoprecipitation (ChIP)

#### Formaldehyde cross-linking and ultrasonic fragmentation

HEK293 cells were cultured in 10 cm dishes and transfected with 10 µg of the E2F1 plasmid in combination with liposome 3000 for 24 h. A final concentration of 1% formaldehyde was added to the medium, after which the cells were incubated for 10–15 min at 37 °C. A final concentration of 0.125 M glycine was added, mixed, and incubated with the cells at room temperature for 5 min. The medium was then aspirated, and the cells were washed twice with ice-cold PBS. The cells were collected via a cell scraper and placed inside 15 ml tubes for centrifugation at 2000 rpm for 5 min. The supernatant was then removed, and the pellets were resuspended in 0.1 ml of 10× cocktail and 0.9 ml of ChIP lysis buffer (50 mM HEPES-KOH, pH 7.5; 140 mM NaCl; 1 mM EDTA, pH 8; 1% Triton X-100; 0.1% sodium deoxycholate; 0.1% SDS) on ice for 30 min. Ultrasonic fragmentation was performed as follows: 50% power, 8 s impact/10 s gap, 5 min. The lysate was then centrifuged at 4 °C for 10 min, after which the supernatant was collected.

#### DNA purification

Then, 50 µl of sonication product was mixed with 70 µl of elution buffer (1% SDS, 100 mM NaHCO3), uncrosslinked overnight at 65 °C with 4·8 µl of 5 M NaCl and 2 µl of RNaseA (10 mg/ml), and then treated with 2 µl of 10 mg/ml proteinase K at 60 °C for 1 h to degrade the impurities. One hundred µl of sample was diluted with 90 µl of TE buffer (10 mM Tris pH 8·0, 1 mM EDTA) containing 200 µl of elution buffer and 2 µl of proteinase K. After mixing well, 400 µl of phenol: chloroform (25:24) and 400 µl of chloroform were added, the mixture was centrifuged at maximum speed, and the supernatant was removed. Then, 900 µl of anhydrous ethanol containing 1 µl of 20 mg/ml glycogen and 50 µl of 4 M LiCl were added, and the mixture was vortexed adequately to precipitate the DNA. The mixture was subsequently centrifuged at maximum speed, after which the precipitate was washed with 1 ml of 75% ethanol. Finally, the tube was inverted to dry the DNA, and 30 µl of water containing 1 µl of RNaseA was added to dissolve the DNA at 37 °C for 1 h.

#### Immunoprecipitation

The magnetic beads were washed 5 times with 200 µl of PBS supplemented with 0·1% Tween 20 and incubated with 200 µl of E2F1 antibody in PBS overnight at 4 °C. The next day, the unbound antibodies and magnetic beads were removed, and 200 µl of sonication product and 800 µl of ChIP RIPA buffer (+ cocktail) were added to the magnetic bead-bound antibodies, which were subsequently stored at 4 °C overnight. The supernatant was discarded, and the samples were washed with a mixture of salt (0·1% SDS, 1% Triton X-100, 2 mM EDTA, 20 mM Tris-HCl (pH 8·0), 150 mM NaCl, high salt (0·1% SDS, 1% Triton X-100, 2 mM EDTA, 20 mM Tris-HCl (pH 8·0), 500 mM NaCl), LiCl wash buffer (0·25 M LiCl, 1% NP-40, 1% sodium deoxycholate, 1 mM EDTA, 10 mM Tris-HCl (pH 8·0)) and TE buffer. Then, 120 µl of elution buffer was added, the mixture was vortexed, the supernatant was transferred to a new tube, and the DNA was purified as described above.

#### PCR

The JASPAR database was designed to provide the best specification of DNA binding profiles for each transcription factor. The promoter sequence of FBXL16 was predicted accurately by GeneCopoeia (https://www.genecopoeia.com/product/search/detail.php?prt=22&cid=&key=HPRM53066&type=promoter&choose=FBXL16). By using JASPAR online analysis, the confidence interval was set at 85%, and three DNA motifs were confirmed to bind to E2F1 in the FBXL16 promoter region. For this reason, primers were designed to validate the motif region of the FBXL16 promoter.

### FBXL16 lentivirus administration

Six-month-old male 3×Tg-AD transgenic mice (Southwest Medical University Laboratory Animal Center) and WT mice (both from the C57BL/6 background) were used and housed under specific pathogen-free conditions. Animal care and experimental procedures were performed in accordance with the Southwest Medical University Animal Care Facility Guidelines. Lentiviral plasmids containing the mouse FBXL16 coding sequence (LV-F16), green fluorescent protein-tagged lentiviral plasmids, and their packaging lentiviral supernatants were purchased from VectorBuilder. Negative control (LV-GFP) plasmids and viral supernatants (LV-F16, 3·33 × 10^8^ TU/ml; LV-GFP, 4·02 × 10^8^ TU/ml) were also obtained. Six-month-old mice were randomly divided into four groups. 3×Tg-AD/LV-F16, 3×Tg-AD/LV-GFP, WT/LV-F16 and WT/LV-GFP (*n* = 10 per group). After anesthetization, the mice were fixed horizontally on a brain stereotaxic instrument, and a 1–1.5 cm incision was made via a scalpel from the midpoint of the line connecting the posterior eye corners of the mice. The skull was then scraped with a peroxide swab to fully expose the fontanelle. The fontanelle was moved back 2 mm, the left and right paracentesis 2 mm, and the depth 3 mm according to the specific coordinates of the injection site. First, the hole was punched with a cranial turn, followed by a slow (at least 1 µl/min) injection of 3 µl of virus supernatant per mouse. Notably, the diffusion time must be greater than the injection time to allow more virus to remain within the hippocampal region before the needle slowly exits. This was followed by trigeminal suture and skin disinfection, after which the mice were allowed to awaken. Three months later, the lentivirus was injected again via the same procedure.

### In vivo live imaging

Two months after the injection of green fluorescent protein-labeled lentiviral plasmids and their packaging lentiviral supernatants in a stereotaxic apparatus, 3×Tg-AD transgenic mice were chosen for detection of the expression of lentivirus-bearing FBXL16 in the hippocampal region via in vivo animal fluorescence imaging techniques.

### Measurement of neurotransmitter levels

Six months after the first injection of FBXL16 lentivirus, the mice were sacrificed and the whole brain was weighed precisely and placed in an ice bath. Prechilled 0.2% formic acid/water was added, and the brain tissue was homogenized by ultrasonication in an ice bath. After mixing 0.2 ml of brain homogenate and 1 ml of prechilled 0.2% formic acid/water, the supernatant was centrifuged and left for mass spectrometric analysis. Five milligrams of each standard control was weighed and dissolved in methanol/water containing formic acid (1:1) to make a stock solution of 1 mg/ml, which was stored in the refrigerator at -80 °C until further use. A TSK Gel amide 80 column (4·5 mm×150 mm, 5 μm) with a column temperature of 35 °C and mobile phase acetonitrile (6 mM ammonium formate aqueous solution [pH = 5·5, 67·5: 32·5]) with a flow rate of 0·2 ml/min (injection temperature of 4 °C) was used. Mass spectrometric ionization via electrospray ionization in positive ion multiple reaction detection (MRM) mode was performed. The ion source and other related parameters were optimized to a spray voltage of 4500 V and a heating temperature of 450 °C.

### Bead-based multiplex assay

The desired beads were vortexed vigorously for 1 min to mix the beads well. The serum from each animal group was diluted two times with assay buffer (50 µl sample + 50 µl of assay buffer). Then, 25 µl of assay buffer, 25 µl of sample, and 25 µl of mixed beads were added to each tube. After incubation at room temperature for 2 h, 25 µl of detection antibody was added to each tube. After incubation at room temperature for 1 h, 25 µl of SA-PE was added to each tube and shaken at 1,000 rpm for 30 min at room temperature before being subjected to centrifugation for 5 min at 1,000 × g. 125 µl of supernatant was aspirated and mixed with 300 µl of 1× wash buffer. The beads were mixed well and transferred to flow-through tubes before further flow analysis (BD Pharmingen, USA). The raw beads were used to set the photomultiplier tube voltage of the regulated channel APC. For experiments running FACSDivaTM, the PE channel was selected for reporting, and the APC channel was selected for bead classification.

### Hematoxylin and eosin (H&E) staining

The whole brains of the mice, including the hippocampi of cornu area 1 (CA1), cornu area 3 (CA3), dentate gyrus (DG), and cortical areas, were assessed pathologically by HE staining. The brain sections were fixed with 4% PFA, soaked in xylene, and then dehydrated in 100%, 95%, 90%, 80%, and 70% alcohol. The sections were then stained with hematoxylin (50 °C) for 30 s, incubated in 1% hydrochloric acid alcohol for 10–20 s, washed with 0·5% ammonia hydroxide for 10 s, stained with eosin for 3–5 s, and finally dehydrated in 70%, 75%, 80%, 90% and 100% alcohol. After transillumination in dimethylbenzene, the sections were sealed with neutral adhesive for microscopic observation. Images were captured via light microscopy (Leica, WZ, GER).

### Nissl staining

The whole-brain region of the mouse hippocampus, including the CA1, CA3, DG and cortical areas, was assessed via Nissl staining. The brain sections from each group were dehydrated in the same way as those used for H&E staining, stained with 1% tartrate for 1 h, washed with distilled water, and separated with 70% alcohol for 1 min. The tissue was then dehydrated with 70%, 75%, 80%, 90% and 100% alcohol. After soaking in dimethylbenzene, the sections were sealed with neutral glue for microscopic observation. Images were captured with a light microscope (Leica, WZ, GER).

### Construction of FBXL16 conditional knockout mice

Transcript information was obtained from NCBI for the FBXL16 gene (Gene ID: 214931), which contains six exons and is located on chromosome 17. The gRNA target site for the FBXL16 gene is located between exon 2 and exon 6. The following sequences were used: FBXL16-sgRNA1, 5’- tgccttggtgaatgggctgctgg-3’; FBXL16-sgRNA2, 5’- ccctggtggaggtcccgcccatc-3’. After vector construction, plasmid preparation and plasmid linearization were performed instead of viral packaging, the linearized DNA was transcribed into RNA with an in vitro transcription kit, and the gRNA was amplified via PCR. The PCR products were transcribed into RNA from the in vitro transcription PCR products. Purified RNA was used for subsequent oosperm injection, after which the RNA was transplanted into surrogate females to breed targeted knock-in F0 progenitor transgenic mice. The FBXL16 flox/+ mice (F0) were initially crossed with wild-type mice to generate FBXL16 flox/+ mice (F1), along with expanded breeding EmXL-Cre mice to obtain enough EmXL-Cre mice. FBXL16 flox/+ mice were crossed to obtain FBXL16 flox/flox mice (F2). FBXL16 flox/flox mice were crossed with Emxl-Cre mice to screen for the genotype of FBXL16 flox/+/EmXL-Cre mice (F3), which were subsequently crossed with FBXL16 flox/+ or FBXL16 flox/flox mice to obtain offspring from FBXL16 flox/flox/Emxl-Cre conditional KO mice (F4, brain-specific knockout mice) and FBXL16 flox/flox mice (experimental control mice), respectively.

## Results

### The levels of FBXL16 and its interacting proteins are related to ubiquitination and cognitive traits in AD models

**Fig. 1:**
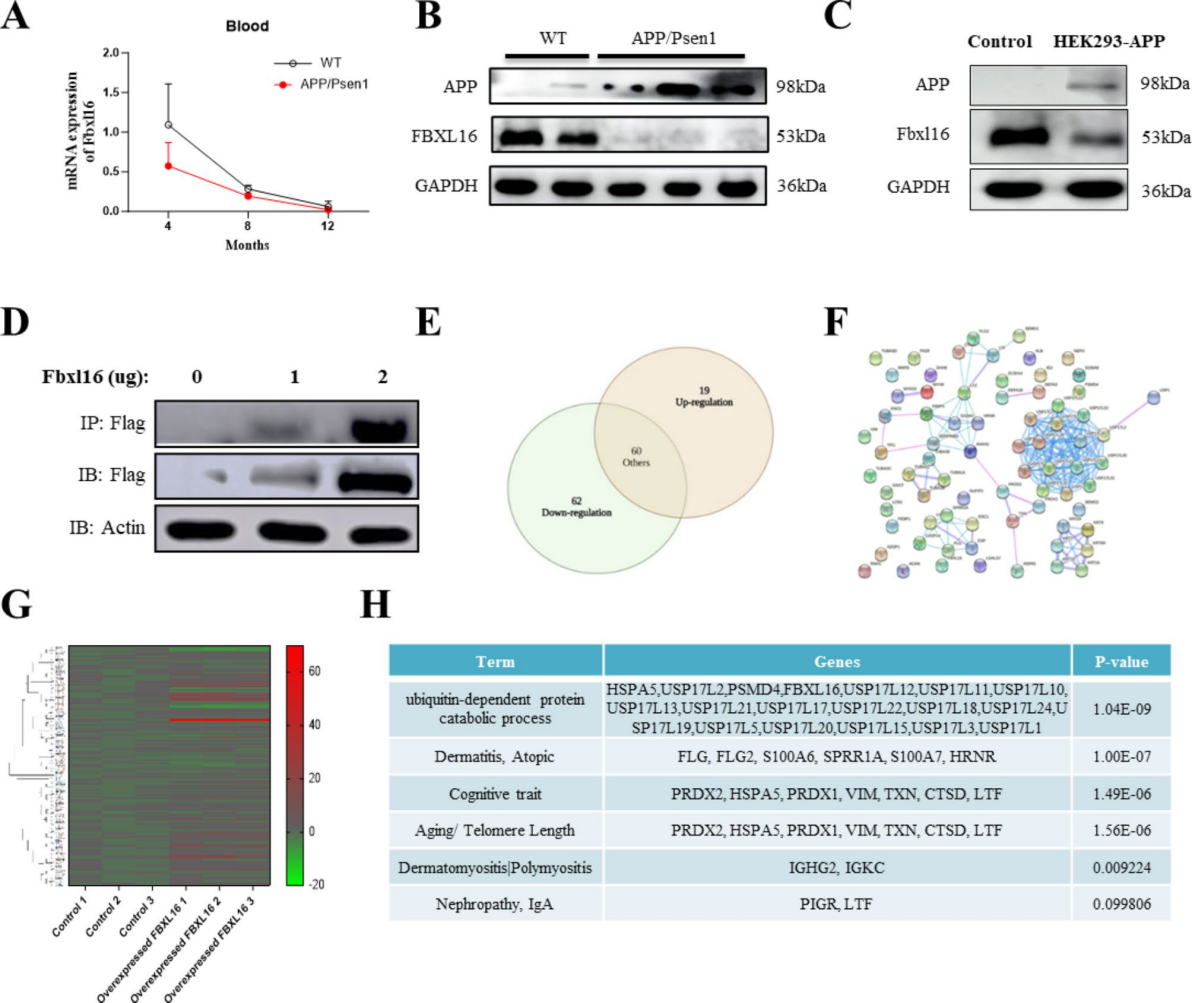
Low expression of FBXL16 in HEK293-APP stably transfected cell line and APP/PSEN transgenic mice. (**A**) Gene expression levels of FBXL16 in an age-dependent manner (4, 8 and 12-month) in APP/PSEN mice (*n* = 5) compared with wild-type mice (*n* = 5). (**B**) Protein expression levels of FBXL16 and APP protein in brain tissues of APP/PSEN mice (*n* = 4) versus wild-type mice (*n* = 6). (**C**) Protein expression levels of FBXL16 and APP protein in HEK293 cells stably expressing APP. (**D**) 1 µg and 2 µg Flag-FBXL16 was overexpressed and subsequently immunoprecipitation-labeled in HEK293 cells. (**E**) Overlaps between differentially expressed genes of the up-regulation and down-regulation in 141 proteins according to NCBI and UniProt databases. (**F**) STRING analysis of these 141 genes. (**G**) Heatmap diagram of these 140 selected genes validated by real time PCR. (**H**) The GAD_DISEASE database showed that these genes were highly associated with ubiquitin-dependent protein catabolic process (1.04E-09), Dermatitis, Atopic (1.00E-07), cognitive traits (1.49E-06) and aging (1.56E-06). The full length of western blotting images was showed in the Supplementary Fig. 1C and 1D

Aged APP/PSEN double transgenic mice are well adopted for AD research because they possess a broad range of behavioral and pathological AD-like phenotypes, such as cognitive and noncognitive deficits, as well as the accumulation of amyloid plaques [16]. To this end, the APP/PSEN mice were firstly adopted in the present study for the evaluation on both spatial memory (Supplementary Fig. 1A) and motor deficits as the mice age increased from 4 to 12 months (Supplementary Fig. 1B). With the reported correlation of decreased levels of ubiquitination-related genes such as FBXL16 in the human mature oocytes of older people [17], the role of FBXL16 and ubiquitination in age-related AD was investigated for the first time in an APP/PSEN mouse model. Figure 1A showed that the mRNA levels in APP/PSEN mice decreased significantly with age compared with those in wild-type mice. Similarly, the protein levels of FBXL16 in the 8-month-old APP/PSEN mice were also lower than those in wild-type mice (Fig. 1B), indicating a potential role for FBXL16 in the development of AD. Consistent with the reduction in the protein level of FBXL16 in the aged mouse brain tissue, stable APP-overexpressing HEK293 cells also presented a decrease in the protein level of FBXL16 (Fig. 1C), suggesting a possible protective role of FBXL16 in the regulation of AD-related disease-related proteins. To confirm the mechanistic role of FBXL16 in AD, overexpression and subsequent immunoprecipitation of flag-tagged FBXL16 were therefore performed in HEK293 cells (Fig. 1D). The final immunoprecipitated protein sample underwent digestion and was analyzed via LC‒MS/MS tandem secondary mass spectrometry. From this analysis, 141 proteins that interact with FBXL16 were identified. Among the proteins analyzed, 19 were found to be upregulated and 62 were downregulated, as shown in Fig. 1E. The genes that were significantly upregulated or downregulated were then input into STRING to create Fig. 1F.

To further verify these results, the SHSY-5Y cell line with overexpressing of FBXL16 was used for qPCR analysis as displayed in Fig. 1G. On the basis of databases such as NCBI, UniProt, GO, and KEGG, the genes were categorized into various groups including “Dermatitis, Atopic,” “Cognitive Trait,” “Aging/Telomere Length,” “Dermatomyositis/Polymyositis,” and “Nephropathy, IgA,” which are listed in Fig. 1H. A potential interaction network and mechanisms of the identified FBXL16-interacting proteins were revealed through STRING functional protein correlation network analysis, which included 15 USP17 family genes (USP17L2, USP17L12, USP17L11, USP17L10, USP17L13, USP17L21, USP17L17, USP17L22, USP17L18, USP17L24, USP17L19, USP17L5, USP17L20, USP17L15, USP17L3, and USP17L1) and two other genes (HSPA5 and PSMD4), along with FBXL16. These genes are associated with the GO term “ubiquitin-dependent protein catabolic process” (1.04E-09). Additionally, the Genetic Association Disease Database (GAD) (https://geneticassociationdb.nih.gov/) provides data linking these genes to diseases or other phenotypes. According to the GAD_DISEASE database, these genes are strongly associated with cognitive traits (1.49E-06) and aging (1.56E-06). Notably, PRDX2, HSPA5, PRDX1, VIM, TXN, CTSD, and LTF are linked to cognitive traits and aging/telomere length (Fig. 1H). These findings suggest that FBXL16 and its interacting candidate proteins may play significant roles in aging-related cognitive traits and ubiquitination, meriting further investigation.

### FBXL16 activity is regulated by the transcription factor E2F1

**Fig. 2:**
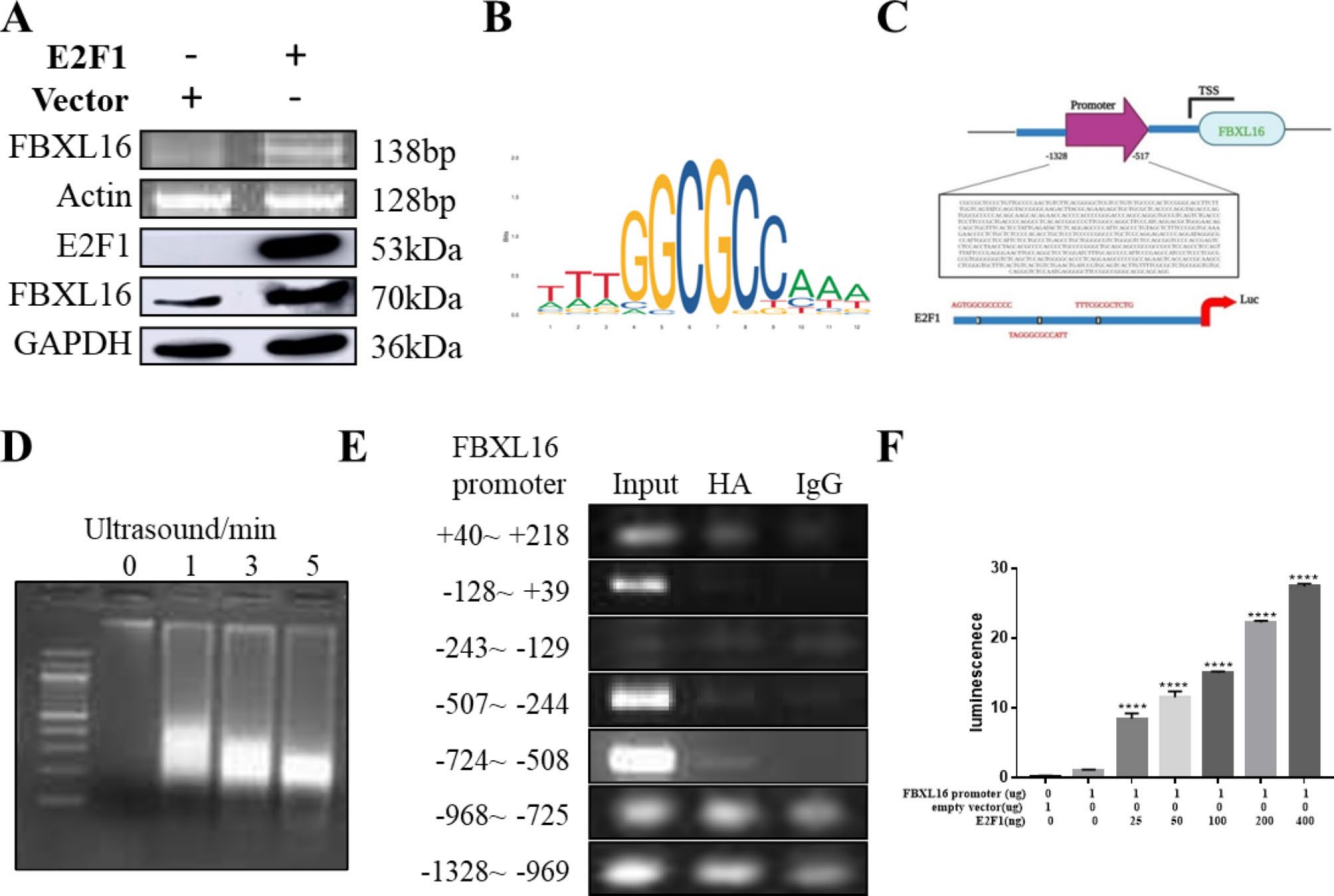
FBXL16 activity is regulated by the transcription factor E2F1. (**A**) Protein expression and gene expression levels of FBXL16 after overexpression of E2F1; (**B**) Predict the DNA-binding region of the E2F1 and FBXL16 promoters (TTTGGCGCCAAA) according to the JASPAR database; (**C**) Three sequences, AGTGGCGCCCCC, TAGGGCGCCATT and TTTCGCGCTCTG, were obtained after analyzing the promoter regions; (**D**) HEK293 cells were transfected with the E2F1and ultrasonic crushing at 50% impulse, 10 s impact/10 s gap and 5 min optimal conditions; (**E**) The core promoter region of the FBXL16 gene is located at -724 ~ -508 bp. (**F**) Luciferase assay of co-transfected promote of FBXL16 and pcDNA3.1-E2F1 (0, 25, 50, 100, 200, or 400 ng) into HEK293 cells. The ratio of firefly luciferase activity to Renilla luciferase activity (reflecting changes in promoter transcriptional activity) was measured 24 h after transfection using the empty vector pEZX-PL01 as a negative control. ***P < 0.001 vs. FBXL16 promoter group

Previous studies have reported that the transcriptional activity of the FBXL protein family is activated by the E2F family [18]. To investigate the role of this transcription factor family in the activity of FBXL16, a plasmid expressing the transcription factor E2F1 was transiently transfected into H4 cells. As shown in Fig. 2A, increased FBXL16 gene and protein expression was detected after the overexpression of E2F1. As a transcription factor, E2F1 needs to bind to the FBXL16 promoter region to activate its expression. The JASPAR database, which is a public database containing information on transcription factors and DNA-binding sites, was used to predict the DNA binding region (TTTGGCGCCAAA) of the E2F1 and FBXL16 promoters (Fig. 2B). After analysis of the promoter regions, 3 sequences, AGTGGCGCCCCC, TAGGGCGCCATT, and TTTCGCGCTCTG, were obtained (Fig. 2C). To confirm this prediction, H4 cells were transfected with the E2F1 plasmid before being subjected to lysis by ultrasonic fragmentation (Fig. 2D). According to the Ch-IP results, as shown in Fig. 2E, the core promoter region of the FBXL16 gene is located at -724 ~ -508 bp, suggesting the potential binding of E2F1 to FBXL16. Moreover, the full-length promoter vector containing the transcription start site (pEZX-PL01-FBXL16) linked to the luciferase reporter was cotransfected with a plasmid expressing the transcription factor (pcDNA3·1-E2F1) into HEK293 cells (Fig. 2F). With the empty vector pEZX-PL01 used as the negative control, the ratio of firefly luciferase activity to Renilla luciferase activity (reflecting the change in promoter transcriptional activity) was measured 24 h after transfection. According to the dual-luciferase reporter assay results, FBXL16 promoter transcription was increased in a dose-dependent manner upon increasing the expression of the transcription factor E2F1 (0, 25, 50, 100, 200, or 400 ng), indicating that the FBXL16 promoter was positively regulated by E2F1.

### FBXL16 enhances the ubiquitination and degradation of APP

**Fig. 3:**
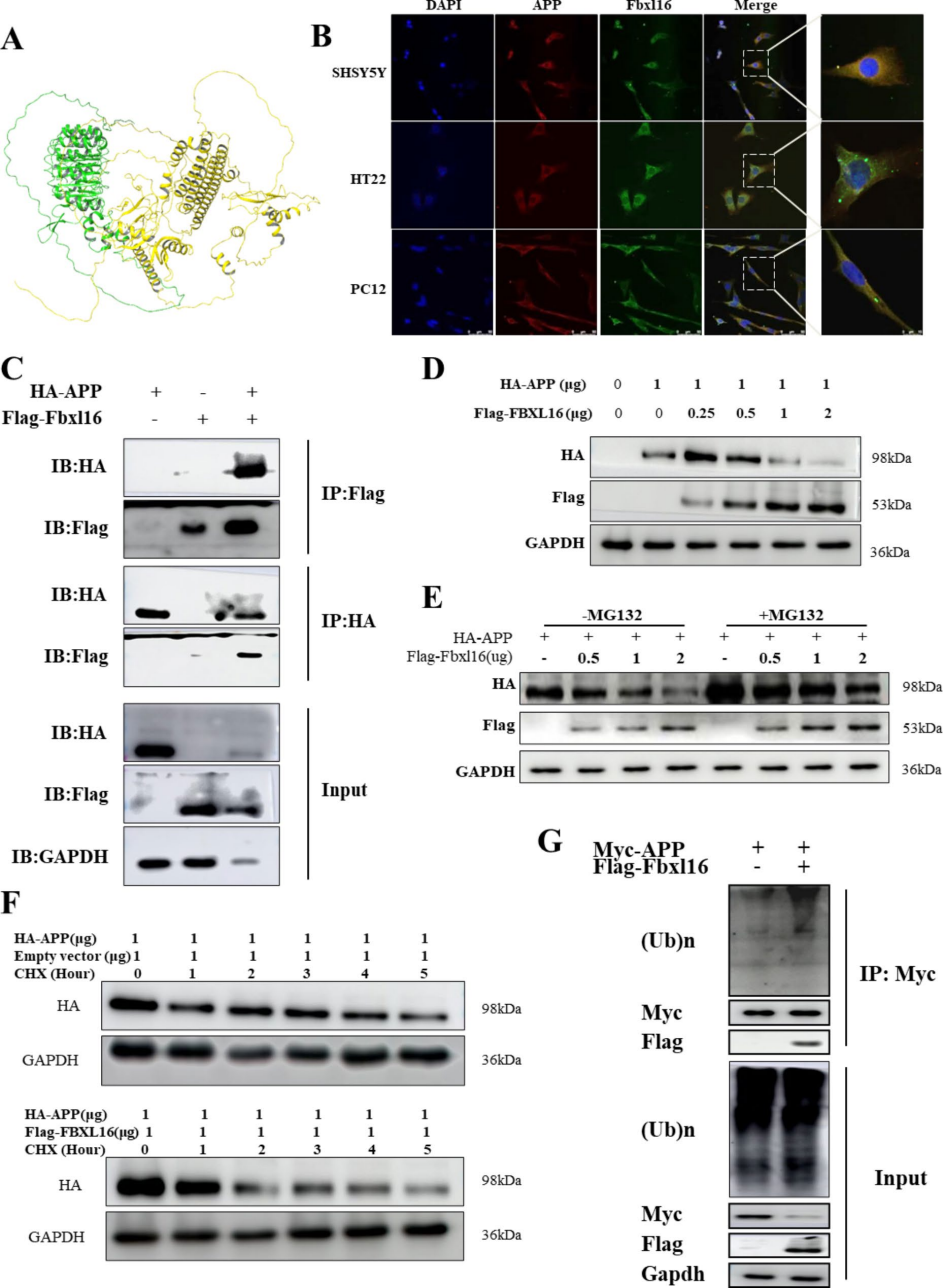
FBXL16 enhances ubiquitination and degradation of APP *in vitro.* (**A**) The corresponding interactions between amino acids of FBXL16 and APP. (**B**) Co-localization of FBXL16 and APP in different neuronal cell types (HT-22, PC-12 and SHSY-5Y cells) was analyzed by fluorescence microscopy. HT-22 cells originate from mice, PC-12 cells from rats, and SH-SY5Y cells from humans. (**C**) Immunoprecipitation of Flag-FBXL16 and Myc-APP plasmids were transfected/co-transfected into HEK293 cells. (**D**) Western blotting of co-transfected Flag- FBXL16 and HA-APP (0, 0.25, 0.5, 1, 2 µg) into HEK293 cells. (**E**) HA-APP was co-expressed with increasing amounts of Flag-FBXL16 protein (0, 0.5, 1, 2 µg) in the presence or absence of the proteasome inhibitor MG132 in HEK293 cells. (**F**) Co-transfected Flag- FBXL16 and HA-APP in the presence of the protein synthesis inhibitor cycloheximide (CHX). (**G**) Immunoprecipitation assay for Myc-APP with or without Flag-FBXL16 in HEK293 cells. (**A**) The full length of western blotting images was showed in the Supplementary Figs. 2–6

With the observation that FBXL16 was downregulated in both stable APP/HEK293 cells and in the brains of APP/PSEN mice, the potential relationship between FBXL16 and APP was explored. First, the protein structures of FBXL16 (*Homo sapiens*) and the neuro-pathogenic protein APP (*Homo sapiens*) were downloaded from the AlphaFold protein structure database (UniProt: Q8N461, P05067) for molecular docking analysis. After that, the two proteins were prepared by using the protein preparation wizard from Schrödinger Maestro. The docking procedures were performed using protein docking modules. Among the 30 binding poses generated, binding pose 3 was found to have the lowest total energy of -41·228 kcal/mol, suggesting that FBXL16 binds to APP. The corresponding interactions between the amino acids in the two proteins are depicted in Fig. 3A. Furthermore, the cellular distributions of FBXL16 and APP in different neuronal cell types (HT-22, PC-12, and SHSY-5Y cells) were analyzed via fluorescence microscopy. Representative images revealed that FBXL16 and APP were colocalized (Fig. 3B) in the cytoplasm. To further validate the direct binding of FBXL16 to APP, Flag-FBXL16 and Myc-APP plasmids were cotransfected into HEK293 cells, and the binding of FBXL16 to APP was confirmed by immunoprecipitation (Fig. 3C). Next, the effect of FBXL16 on APP levels was evaluated via western blotting after cotransfection. The results revealed that increased FBXL16 expression significantly decreased the protein level of APP in a dose-dependent manner (Fig. 3D and Supplementary 2 A).

Since FBXL16 is a member of the E3 ubiquitin ligase family, the downregulation of APP by FBXL16 via ubiquitination was further investigated. Initially, HA-APP was coexpressed with increasing amounts of the Flag-FBXL16 protein in HEK293 cells in the presence or absence of the proteasome inhibitor MG132 (Fig. 3E). Immunoblotting revealed that in the absence of MG132, the HA-APP protein level decreased with increasing Flag-FBXL16. However, the HA-APP protein level increased significantly in the presence of MG132, suggesting that the proteasome plays a role in APP degradation. In a parallel experiments in the presence of the protein synthesis inhibitor cycloheximide (CHX), the overexpression of FBXL16 also accelerated the degradation of HA-APP (Fig. 3F). Compared with that in mock-transfected cells, the reduction in APP expression was greater in FBXL16-transfected cells, suggesting the involvement of FBXL16 in the degradation of APP. Furthermore, the results of the ubiquitination of APP detected by an immunoprecipitation assay revealed that FBXL16 significantly promoted the ubiquitination of the APP protein (Fig. 3G). Taken together, these findings suggest that FBXL16 may enhances the proteasomal degradation of APP via ubiquitination.

### FBXL16 protein overexpression improves the cognitive behavior of 3×Tg-AD mice

**Fig. 4:**
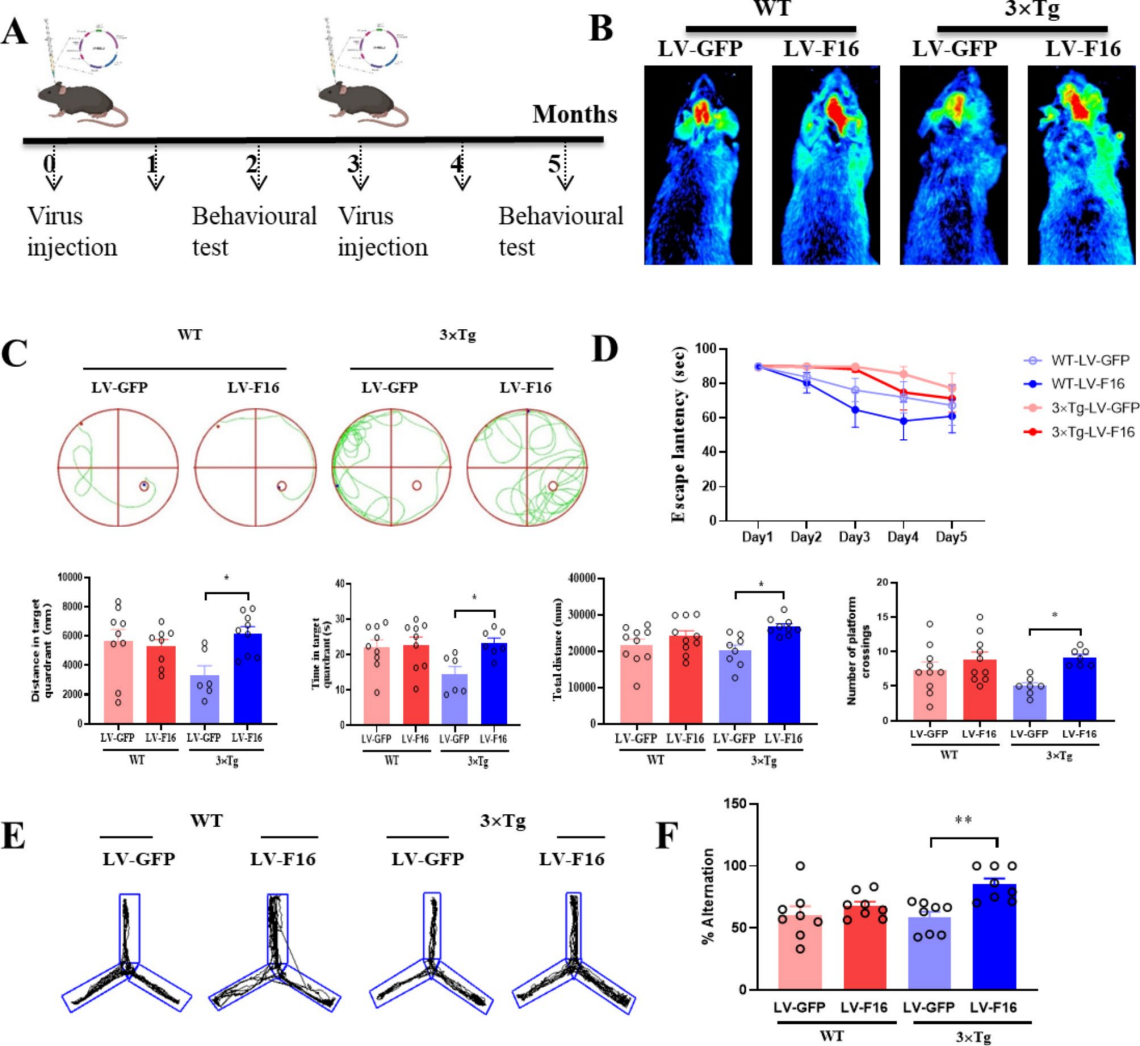
Overexpression of FBXL16 Alleviates Cognitive deficits in 3×Tg AD Mice. (**A**) Schedule for animal experiments. (**B**) Fluorescence imaging in vivo. (**C**, **D**) Representative images depict the swimming paths of mice during the performance of the Morris Water Maze. The data includes the number of platform crossings, the distance traveled, and the time spent in the target quadrant. *P < 0.05 vs. LV-GFP in 3×Tg group. (**E**, **F**) Representative tracks and quantification of the percentage of alternation in mice during the Y-maze experiment. *P < 0.01 vs. LV-GFP in 3×Tg group

In addition to the APP/PSEN mice, five-month-old 3×Tg AD mice were adopted for further investigation. The 3×Tg mice were randomly divided into 4 groups: 3×Tg/LV-GFP, 3×Tg/LV-FBXL16, WT/LV-GFP, and WT/LV-FBXL16. A bilateral intracerebroventricular (i.c.v.) injection of the corresponding LV-FBXL16 or LV-GFP control (3 µl per side, 4·10 × 107 TU/ml) was conducted. The second dose of lentivirus was injected in combination with the same treatment and given again after 3 months. The behavioral experiments included water maze and Y-maze tests and were performed according to the schedule shown in Fig. 4A. To confirm the successful injection of the lentiviral expression vector LV-FBXL16 into the mice, the distribution of the real-time fluorescent signal was detected via using an in vivo fluorescence imaging system (Fig. 4B), and representative images were captured. With no experimental mouse death during the injection process, the results revealed a strong fluorescent signal in the mouse brain region overexpressing lentivirus (LV-FBXL16 or LV-GFP control) following stereotaxic brain injection.

Two months after the FBXL16 protein was overexpressed in the hippocampus, a Morris water maze behavioral test was performed to evaluate the learning and memory ability of each group of mice. During the orientation navigation phase of the Morris water maze test, the latency to explore the platform was progressively reduced in all groups of experimental mice over the course of 5 days of training. However, the latency to escape was significantly shorter in the LV-FBXL16 group than in the LV-GFP group for both the wild-type and transgenic AD mice. In terms of spatial exploration ability, compared with GFP control transgenic AD mice, transgenic AD mice overexpressing FBXL16 presented an increased number of platform crossings, distance traveled and time spent within the destination quadrant. (Fig. 4D). Similarly, as revealed by the Y-maze test results, FBXL16 overexpression increased the percentage of spontaneous alternation in transgenic AD mice (Fig. 4E and F), confirming that FBXL16 is protective against hippocampus-dependent spatial learning memory in an in vivo model of AD.

### FBXL16 attenuated histopathological changes in the cortex and hippocampus of 3×Tg-AD mice

**Fig. 5:**
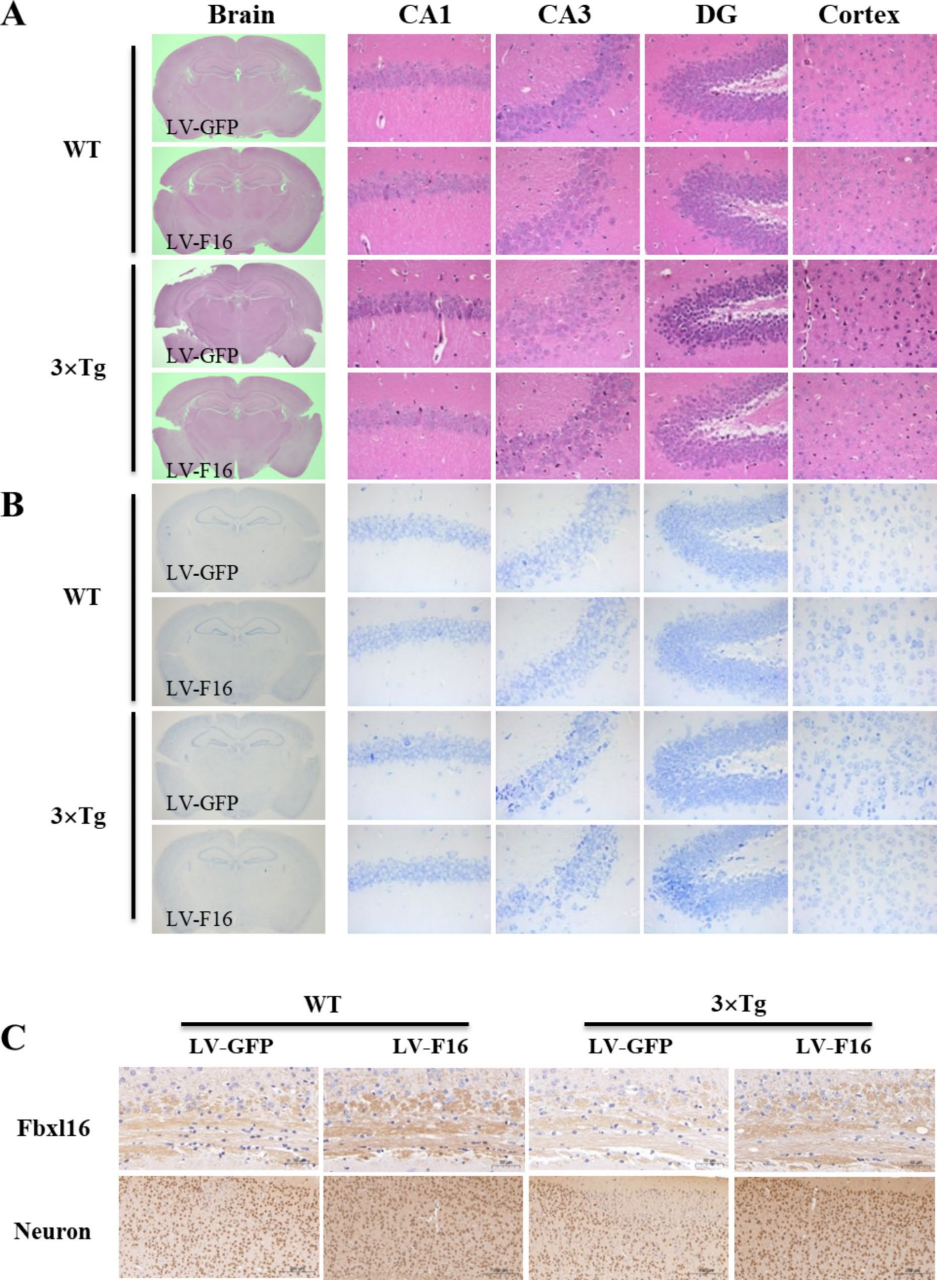
FBXL16 attenuates histopathological changes in the cortex and hippocampus of 3×Tg AD mice with overexpression of LV-FBXL16 by stereotaxic injection. (**A**) Representative images of H&E staining in the cortex and hippocampal CA3, CA1, DG regions. (**B**) Representative images for Nissl staining in the cortex and hippocampal CA3, CA1, DG regions. (**C**) FBXL16 and neurons in the brain sections were identified using the immunohistochemistry method. Representative images were captured using a microscope. Scale bar: 200 μm

Abnormal morphological changes and neuronal loss are pathological features of Alzheimer’s disease. Figure 5A shows representative micrographs of HE-stained images of the cortex and hippocampus from different groups of WT and 3×Tg mice for examination of the histological structure of cells in brain tissue samples. In the cortex and hippocampal CA1, CA3, and DG regions of the WT LV-GFP group, neuronal cells with intact clear and regular cytoarchitectural morphology were characterized by a dense and neat arrangement. However, irregularly shaped and disorganized neuronal cells were observed in the 3×Tg LV-GFP group. In contrast, the pathological status of neuronal cells in the cortex and hippocampal region was improved in the 3×Tg LV-F16 group compared with the 3×Tg LV-GFP control group. Moreover, the morphology, size, and distribution of nerve cells can be clearly visualized via Nissl staining, which is well-adopted for visualizing the structure of nervous tissue, cell type, and cell density. Most of the pyramidal cells in the cortex and hippocampal CA1, CA3, and DG regions of the WT LV-GFP group were regular and tightly packed with clear nucleolar rims, whereas sparsely arranged and vaguely demarcated conical cells were observed in the 3×Tg LV-GFP control group. Notably, neuronal morphology and staining were improved in the 3×Tg LV-FBXL16 group compared with the model group (Fig. 5B). These results confirmed that overexpression of FBXL16 attenuated the histopathological changes in brain tissue. Neurons transmit signals to the brain via receptor synapses and have conductive switching and memory functions, and an increase in neurons improves cognition and memory [19]. Figure 5C showed that, compared with those in the WT LV-GFP group, the 3×Tg LV-GFP group had fewer neurons, whereas the number of neuronal cells was greater in the 3×Tg LV-FBXL16 group than in the 3×Tg LV-GFP group (Supplementary 7B). Taken together, these findings showed that FBXL16 improved neuronal cell density and attenuated histopathological conditions in a transgenic AD mouse model.

### FBXL16 improve neuroinflammation in the 3×Tg AD mice brain overexpressed with LV-FBXL16

**Fig. 6:**
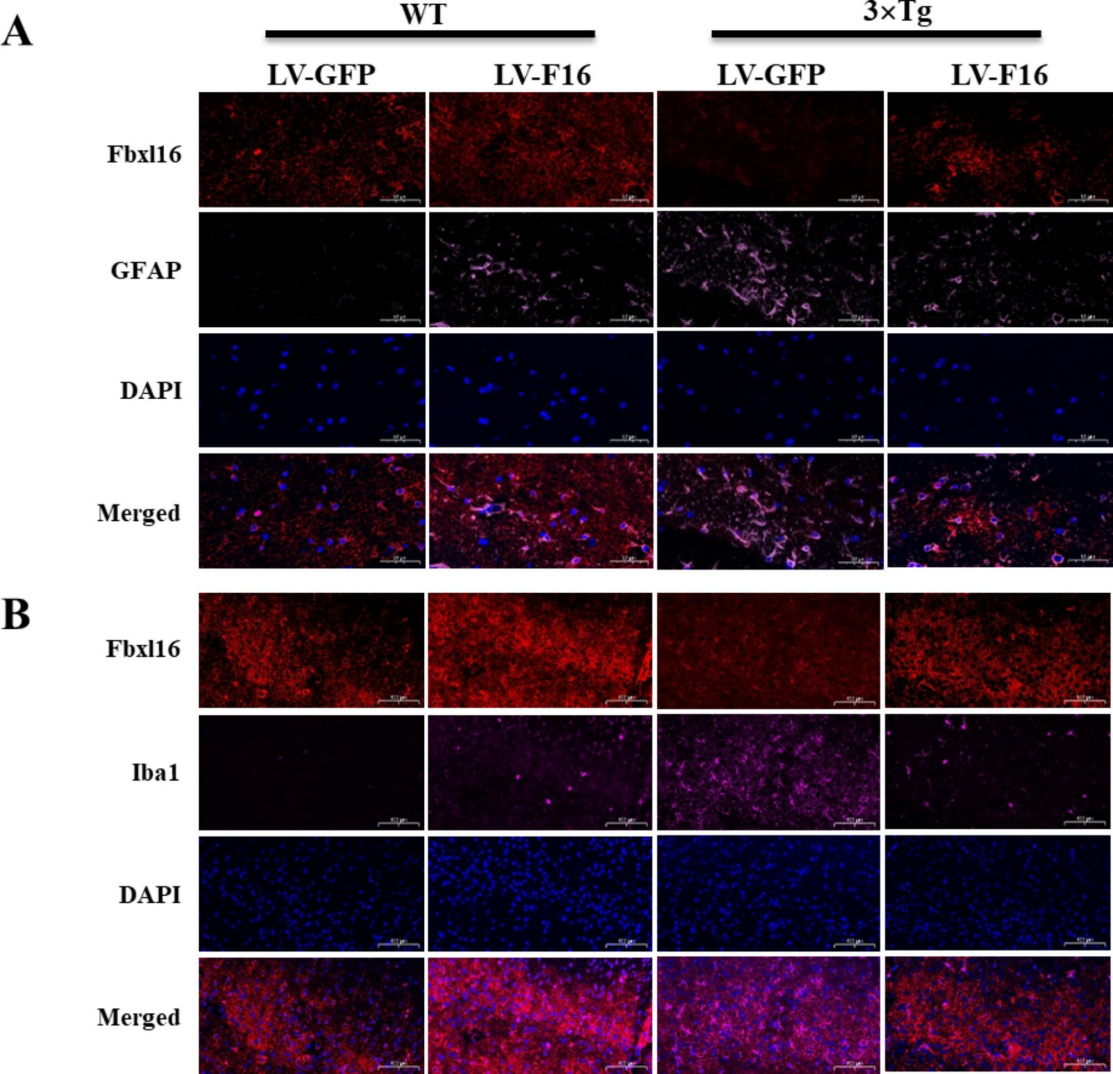
FBXL16 Suppresses Inflammation in the 3×Tg AD mice brain overexpressed with LV-FBXL16. (**A**, **B**) Representative images of cortex regions of the brains in wild-type (WT) and 3xTg-AD mice. FBXL16 positive cells (red color), GFAP positive cells (pink color), Iba1 positive cells (rose red color) and DAPI (blue colour) were stained by corresponding Antibodies. Scale bars = 50 μm

Astrocytes (GFAP) and microglia (Iba1) are important inflammatory indicators that play different roles in the inflammatory process of glial cells in the central nervous system. When the nervous system is damaged, infected, or otherwise stimulated, an inflammatory response is triggered in which astrocytes and microglia are involved is triggered [20]. During inflammation, astrocytes and microglia release a range of cytokines and chemical mediators, such as interleukin-1β (IL-1β), tumor necrosis factor-α (TNF-α) and interleukin-6 (IL-6) [21]. The release of these cytokines can cause the spread and intensification of the inflammatory response, which may negatively affect the nervous system. As shown in Fig. 6A and B, FBXL16 inhibited the activation of astrocytes and microglia in the 3×Tg LV-F16 group compared with the 3×Tg LV-GFP group, suggesting that FBXL16 plays a protective role in suppressing the overactivation of astrocytes and microglia in AD mice. Neurotransmitters are chemicals that transmit signals between neurons or between neurons and effector cells, protect nerve cell membrane structures, and restore cell membrane function under stabilized neurotransmitter levels [22]. The modulation of neurotransmitter levels is widely adopted in the treatment of neuronal diseases such as ischemic stroke, traumatic brain injury, cognitive impairment, and depression [23]. Acetylcholine is an important neurotransmitter in the central cholinergic system that helps individuals maintain consciousness and plays an important role in learning and memory [24]. Aspartic acid facilitates neurogenesis, learning, and neuropathology [25]. In the present study, the levels of these 3 neurotransmitters were determined in the whole brains of WT and 3×Tg mice via high-pressure liquid chromatography. However, while only achylcholine was mildly increased in the 3×Tg LV-F16 group when compared with the other treatment groups, glutamate and aspartic acid levels were not affected (Supplementary Fig. 7A). Taken together, these findings showed the overexpression of FBXL16 may not affect the level of neurotransmitters in the brain.

### FBXL16 improved cognition via ubiquitination-dependent degradation of APP

**Fig. 7:**
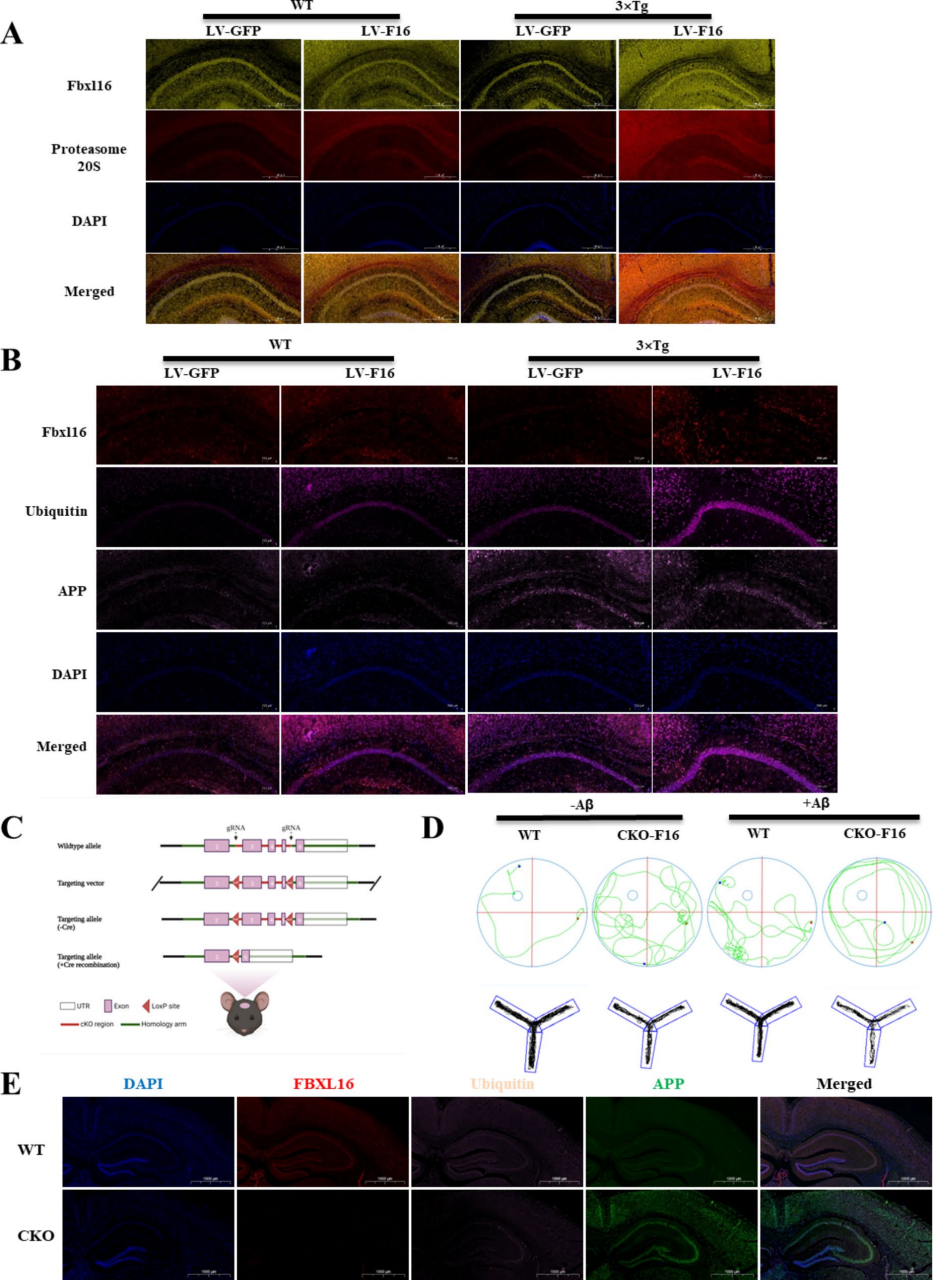
FBXL16 Improves cognitive performance of 3×Tg AD mice brain overexpressed with LV-FBXL16. (**A**) Representative images of hippocampal regions of the brains in WT and 3×Tg-AD mice. Proteasome 20 S positive cells were stained by anti-Proteasome 20 S antibody (red color), while FBXL16 were stained with anti-FBXL16 antibodies (green color). Scale bars = 500 μm. (**B**) Representative images of hippocampal regions of the brains in wild-type (WT) and 3×Tg-AD mice. FBXL16 positive cells were stained by anti-FBXL16 antibody (red color), Ubiquitin positive cells were stained by anti-Ubiquitin antibody (rose red color), APP positive cells were stained by anti-APP antibody (pink color), DAPI (blue colour) was applied for nuclear counterstaining. Scale bars = 500 μm. (**C**) FBXL16 conditional knockout mice were generated via CRISPR/Cas9 technology. (**D**) Representative images on the swimming tracks of mice when the water maze and Y maze were perfomed. (**E**) Representative images of hippocampal regions of the brains in WT and FBXL16 cko mice. Scale bars = 1000 μm

While FBXL16 promoted the proteasomal degradation of APP via ubiquitination, the possible role of FBXL16 in APP degradation in vivo was further investigated. Immunohistochemistry experiments demonstrating the increased intensity of proteasomal markers (Proteasome 20 S) suggest a potential enhancement in ubiquitin-dependent APP degradation following the overexpression of LV-FBXL16 in AD mice, as depicted in Fig. 7A. As shown in Fig. 7B, ubiquitination increased significantly in the mouse brain region overexpressing LV-FBXL16, whereas the level of APP decreased compared with that of the LV-GFP control, indicating that FBXL16 is necessary for ubiquitination-mediated APP clearance. To confirm the necessity of FBXL16 for the ubiquitination of APP, FBXL16-cko mice were first generated via CRISPR/Cas9 technology. This involved the electrotransfer of fertilized eggs with specifically designed sgRNAs and ssDNA (Fig. 7C). Interestingly, compared with the control mice, FBXL16-cko mice with Aβ overexpression, which were induced via stereotaxic injection, presented more severe cognitive impairment in water maze and Y maze tests (Fig. 7D). These findings suggest that FBXL16 plays a crucial role in maintaining proper cognitive and memory function in the AD disease mouse model. In line with the results observed following the injection of LV-FBXL16, Fig. 7E indicates that APP levels were significantly higher in the FBXL16-cko group than in the control group, in which Aβ was overexpressed. This finding provides further evidence supporting the role of ubiquitin-dependent APP degradation after overexpressing LV-FBXL16 in AD mice.

## Discussion

AD is a devastating neurodegenerative disorder that affects millions of people worldwide [26]. Its etiology is not fully understood, but studies have identified several factors that contribute to its development. One interesting candidate implicated in AD is the family of F-box proteins. F-box proteins belong to a large class of proteins that are involved in protein degradation through the ubiquitin-proteasome system. These proteins are characterized by the presence of a conserved F-box domain, which binds to specific substrate proteins. F-box proteins act as adaptor molecules, recruiting other proteins, such as Skp1, Cullin, and Rbx1 (SCF complex), to target proteins for degradation [27]. Several F-box proteins have been linked to neurodegenerative diseases. One of the most studied F-box proteins in this context is Fbxo7. Fbxo7 interacts with proteins involved in Parkinson’s disease, such as PARKIN15 [28]. In the context of neurodegenerative diseases such as AD, disruptions in protein homeostasis and the accumulation of misfolded proteins are common features [29]. The ubiquitin-proteasome system is essential for clearing misfolded or aggregated proteins and preventing their accumulation, which is particularly important for maintaining the health of neurons [30]. In fact, F-box and leucine-rich repeat protein 2, a component of the E3 ubiquitin ligase complex, regulates the metabolism of APP through APP ubiquitination [31]. Unlike other F-box proteins, including FBW7 and SKP2 (FBXL1), FBXL16 does not interact with CUL1 because of its inability to form a functional SCF-E3 ubiquitin ligase complex [32]. Surprisingly, FBXL16 binds protein phosphatase 2 A (PP2A), which contains a B55-specific subunit, and inhibits its substrate vimentin activity to modulate the genesis of FLK1 + progenitor cells [14]. The present study revealed that the expression of FBXL16 decreases in the brains of APP/PSEN double transgenic mice, suggesting that a reduction in FBXL16 expression contributes to the dysregulation of protein homeostasis in neurons, potentially leading to the accumulation of abnormal proteins associated with AD. Previous studies revealed that E2F1 upregulated the transcription of FBXL16 [18]. In the present study, through the use of the JASPAR and TCGA databases, FBXL16 was further shown to be regulated by the transcription factor E2F1, which binds well to the FBXL16 promoter in the core promoter region located at -724 ~ -508 bp. This study has therefore revealed the possible role and mechanism of the F-box protein FBXL16 in the context of disease protein degradation.

Currently, the exact mechanisms by which F-box proteins contribute to AD are still under investigation. One proposed mechanism involves the regulation of proteins involved in tau phosphorylation. Abnormal tau phosphorylation is a hallmark of AD and has been linked to the formation of neurofibrillary tangles. F-box proteins may target tau proteins for degradation, thereby preventing their aggregation and toxicity. The discovery of F-box proteins as key players in AD has opened up new avenues for therapeutic intervention. Inhibition of F-box proteins or targeting of their interaction partners may disrupt the degradation pathway, leading to the accumulation of abnormal proteins and neurotoxicity. Researchers are actively exploring the development of inhibitors or small molecules that interfere with F-box protein function as potential treatments for AD. In recent years, with the rapid development of molecular biology and medical bioengineering, gene therapy and cell replacement therapy, they have attracted increasing amounts of attention and brought hope for the human treatment of AD and other complex diseases [33]. Many studies have confirmed that the injection of neurotrophic factor-related genes or neural stem cells into the ventricles or hippocampi of experimental animals in a stereotactic manner can significantly enhance the spatial learning and memory ability of AD animal models [34]. Based on these reports, the lentiviral FBXL16 gene was used to construct highly stable viral particles via molecular biology and viral packaging techniques. By taking advantage of the wide host range of the lentiviral vector, this gene has the ability to achieve long-term stable expression, good safety and a low immune response [35]. In this study, a lentiviral expression vector was injected into the hippocampus of 3×Tg AD model mice via a stereotaxic method to validate the potential gene targets for future AD therapy. By regulating different factors in the APP metabolic pathway, our study confirmed the role of FBXL16 in improving cognitive and memory impairments in AD. The use of a combination of CRISPR and Cas9 to generate genetically engineered FBXL16-cko mice in this study provides a consolidate way to determine whether F-boxes and E3 ubiquitin ligases regulate APP metabolism through APP ubiquitination.

In the AD model mice, the overexpression of FBXL16 facilitated the ubiquitination-dependent breakdown of APP, thereby reducing Aβ accumulation and its associated neurotoxicity. This reduction in Aβ levels could lead to an improvement in hippocampal function and hence, enhanced spatial learning and memory as evidenced by increased spontaneous alternation behavior. However, the lack of a similar improvement in WT mice might indicate that in the absence of AD pathology, FBXL16 overexpression does not significantly impact APP processing or other pathways involved in cognitive function. This could be due to the tightly regulated nature of ubiquitination processes under normal physiological conditions, where altering the expression of a single ubiquitin ligase such as FBXL16 might not have a noticeable effect. The greater alteration observed in AD mice than in WT mice, regardless of whether they were injected with LV-GFP or LV-FBXL16, this might suggest that the presence of AD pathology, and the subsequent intervention through FBXL16 overexpression create a condition in which the potential for cognitive improvement is more substantial than that in normal aging mice.

**Fig. 8:**
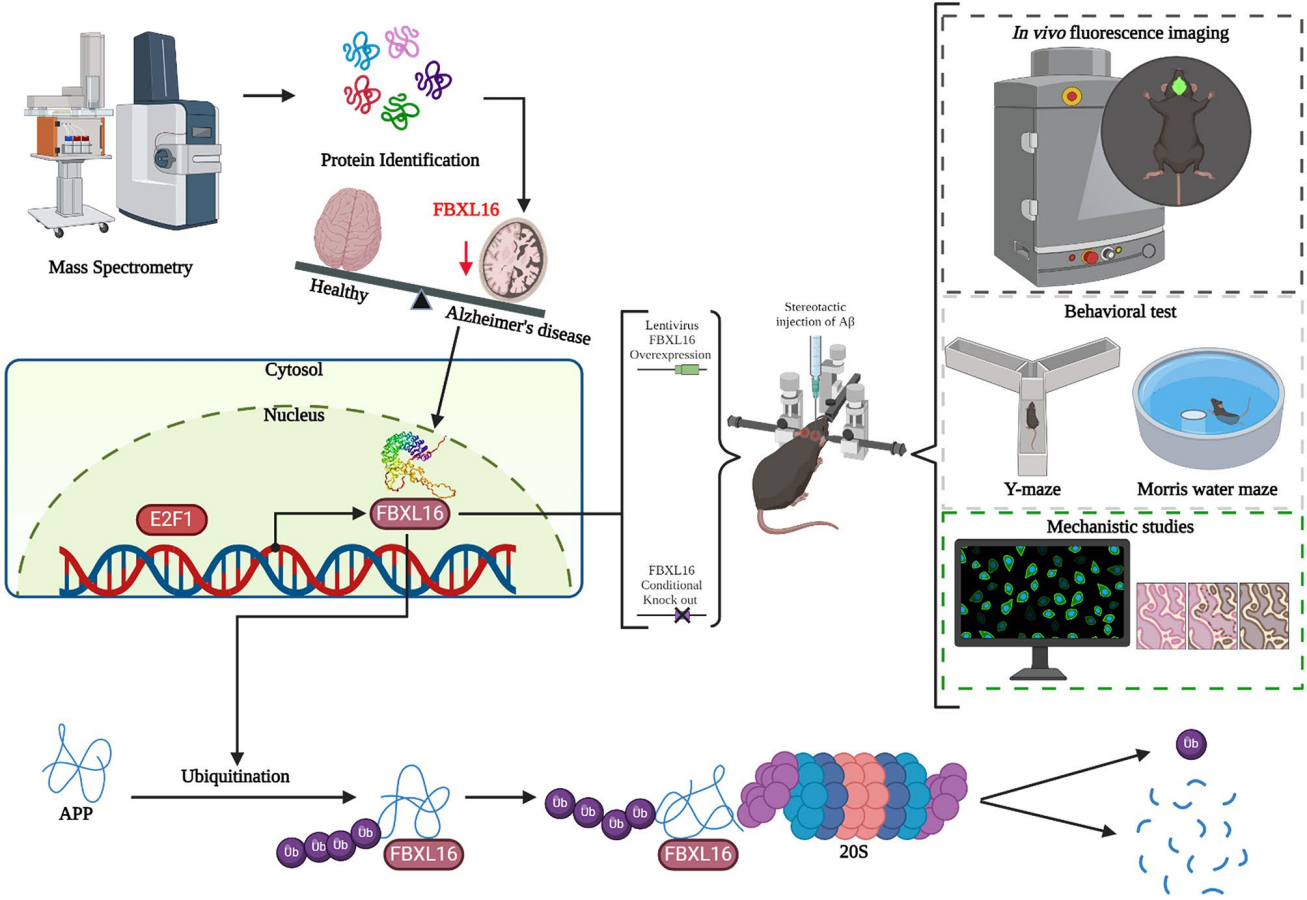
Schematic diagram illustrating the potential functional role of FBXL16 in the ubiquitination-dependent degradation of APP. The level of FBXL16 in the brains of transgenic APP/PSEN mice decreased with age. Proteomic analysis using protein lysates from HEK293 cells overexpressing FBXL16 was conducted to identify potential proteins interacting with FBXL16 related to APP protein ubiquitination. Subsequently, further validation of cognitive ability was performed through lentiviral overexpression of FBXL16 in FBXL16 cko mice and 3x Tg AD mice. E2F1, E2F transcription factor 1. Ub, Ubiquitination. 20 S, Proteasome 20 S

One limitation of our study was the lack of validation of the expression of FBXL16 in both healthy individuals and AD patients, which is a point for future investigations. In addition, owing to the limited availability of hippocampal tissue from mouse models, IHC and ICC, which requires less starting material than do biochemical methods such as pull-down and immunoblotting, was adopted in the present study. One of the primary advantages of ICC or IHC is its ability to provide spatial resolution, enabling the observation of the specific localization of APP and its ubiquitination within intricate structures of the brain, notably in the hippocampus. This method not only preserves the overall architecture of brain tissue but also offers detailed insights into the cellular and subcellular distributions of APP and its modifications. Such information is critically important in the study of neurodegenerative diseases such as AD, as it allows for the identification of regional patterns and can provide valuable clues about disease mechanisms and progression. Additionally, if E2F1 acts as the promoter of FBXL16, whether E2F1 affects the degradation of APP via FBXL16 still warrants further investigation. Taken together, these findings confirmed the novel role of FBXL16 in the ubiquitinated degradation of APP in vivo (Fig. 8).

## Conclusion

The role of F-box family proteins in AD is complex and multifaceted. Through their interaction with substrate proteins, F-box proteins contribute to the degradation of proteins involved in AD pathogenesis. The involvement of these proteins in the possible regulation of tau phosphorylation and APP processing suggests a potential therapeutic opportunity for targeting these proteins for the treatment of AD. Further research is necessary to fully understand the mechanisms and therapeutic potential of F-box proteins in this devastating disease. The current study explored how FBXL16 lowers APP to ameliorate symptoms of AD. FBXL16 is positively regulated by the transcription factor E2F1, which directly binds to its promoter. Furthermore, cognitive performance in the 3×Tg-AD mouse model improved when FBXL16 was overexpressed via lentivirus. An FBXL16-cko-AD mouse model was developed via stereotaxic injection of Aβ into the brain to evaluate the physiological effects of FBXL16 deficiency. Our study suggest the role of FBXL16 in the ubiquitination-dependent degradation of APP, which has not been extensively explored in previous studies. This finding opens new avenues for understanding the molecular mechanisms underlying AD and suggests potential therapeutic targets that have not been considered before.

## Electronic supplementary material

Below is the link to the electronic supplementary material.

Supplementary Material 1

## Data Availability

Data will be made available on request.

## Abbreviations

Aβ: β-amyloid
AD: Alzheimer’s disease
APP: Amyloid precursor protein
CA1: Cornu area 1
CA3: Cornu area 3
ChIP: Chromatin immunoprecipitation
CHX: Cycloheximide
cko: Conditional knockout
Co-IP: Coimmunoprecipitation
DG: Dentate gyrus
FBXL16: F-box and leucine-rich repeat protein 16
GAD: Genetic Association Disease Database
H&E: Hematoxylin and eosin
i.c.v.: Intracerebroventricular
IL-1β: Interleukin-1β
IL-6: Interleukin-6
LV-F16: Lentiviral plasmids containing the mouse FBXL16 coding sequence
MRM: Mass spectrometric ionization via electrospray ionization with positive ion multiple reaction detection
NFTs: Neurofibrillary tangles
PP2A: Protein phosphatase 2 A
SDS‒PAGE: SDS‒polyacrylamide gel
TNF-α: Tumor necrosis factor-α
UPS: Ubiquitin‒proteasome system
