# Supplementary material for: FBXL16: a new regulator of neuroinflammation and cognition in Alzheimer’s disease through the ubiquitination-dependent degradation of amyloid precursor protein
Source: Biomark Res. 2024 Nov 21;12:144. doi: 10.1186/s40364-024-00691-w (PMC11580471; doi:10.1186/s40364-024-00691-w)

Supplementary Figure 1

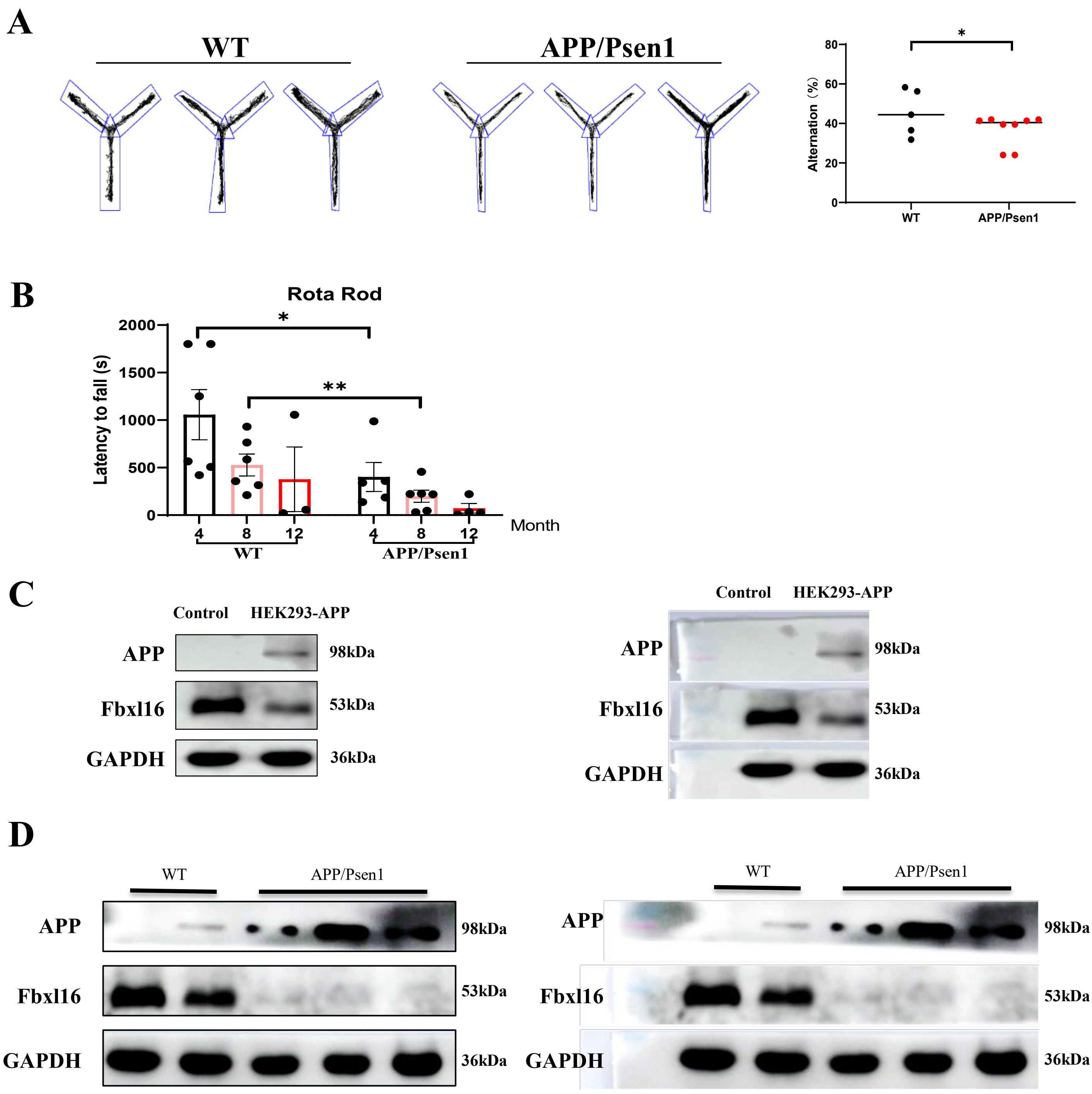

Supplementary Figure 2

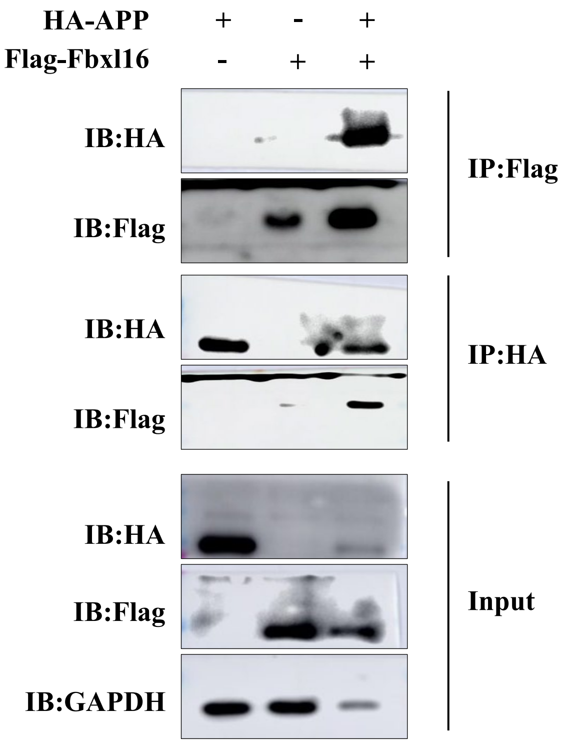

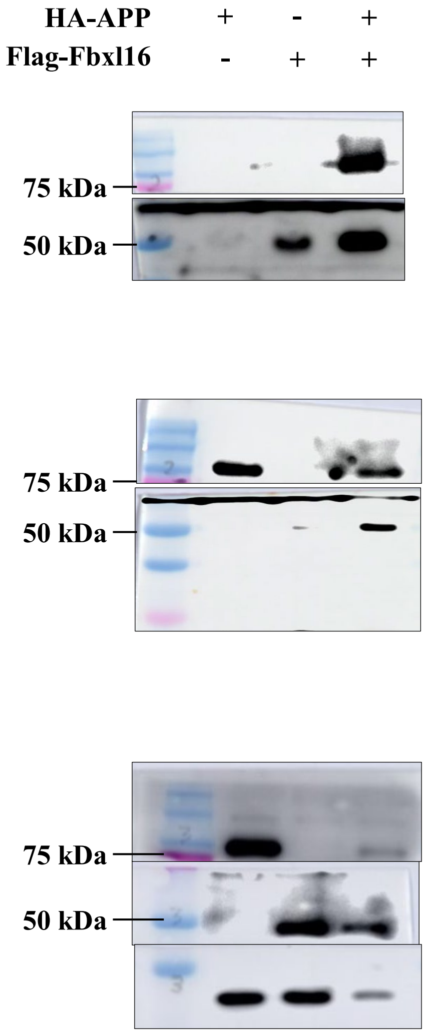

Supplementary Figure 3

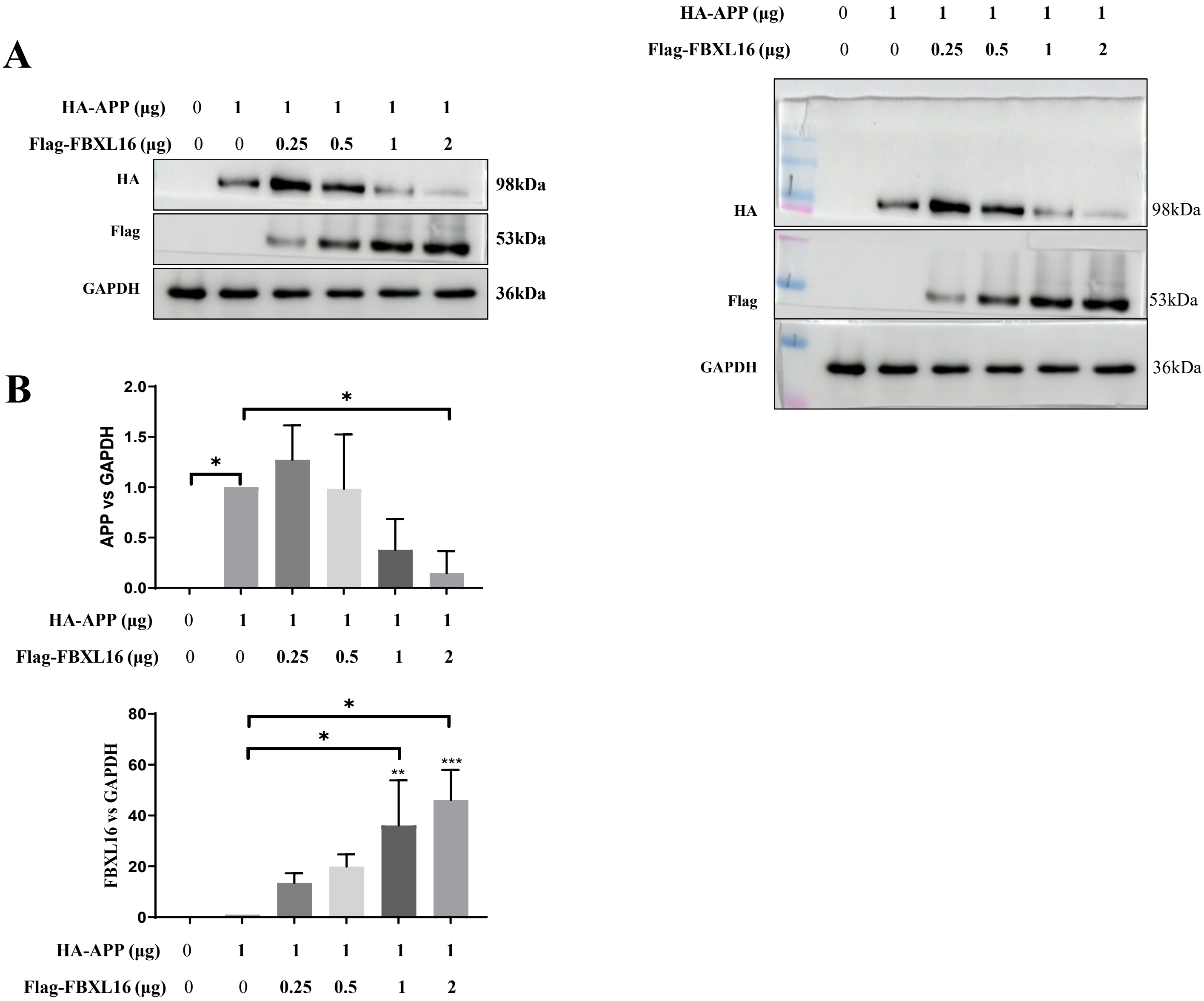

Supplementary Figure 4

A

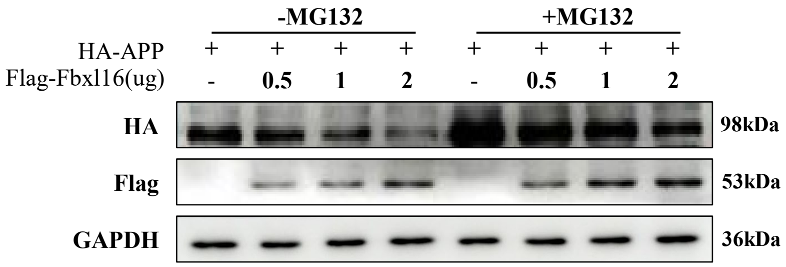

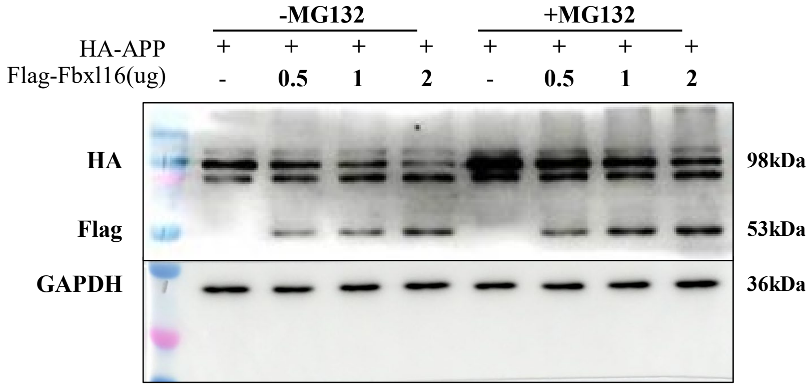

B

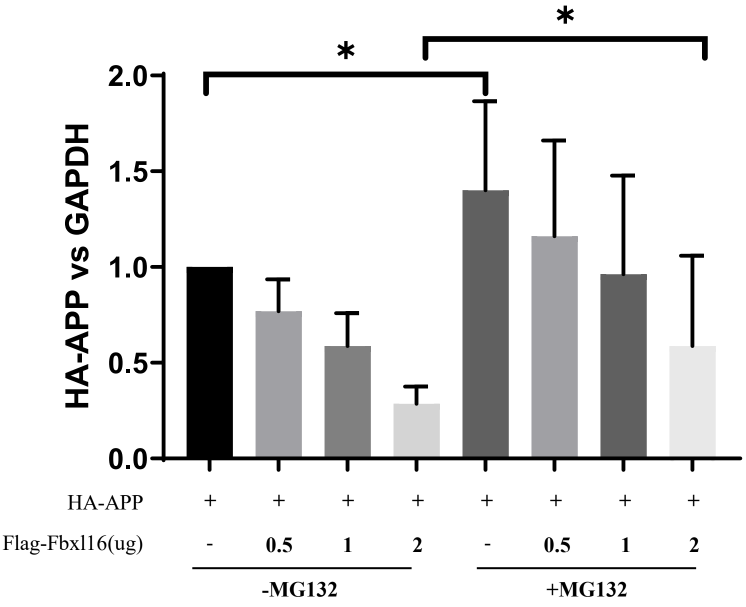

Supplementary Figure 5

A

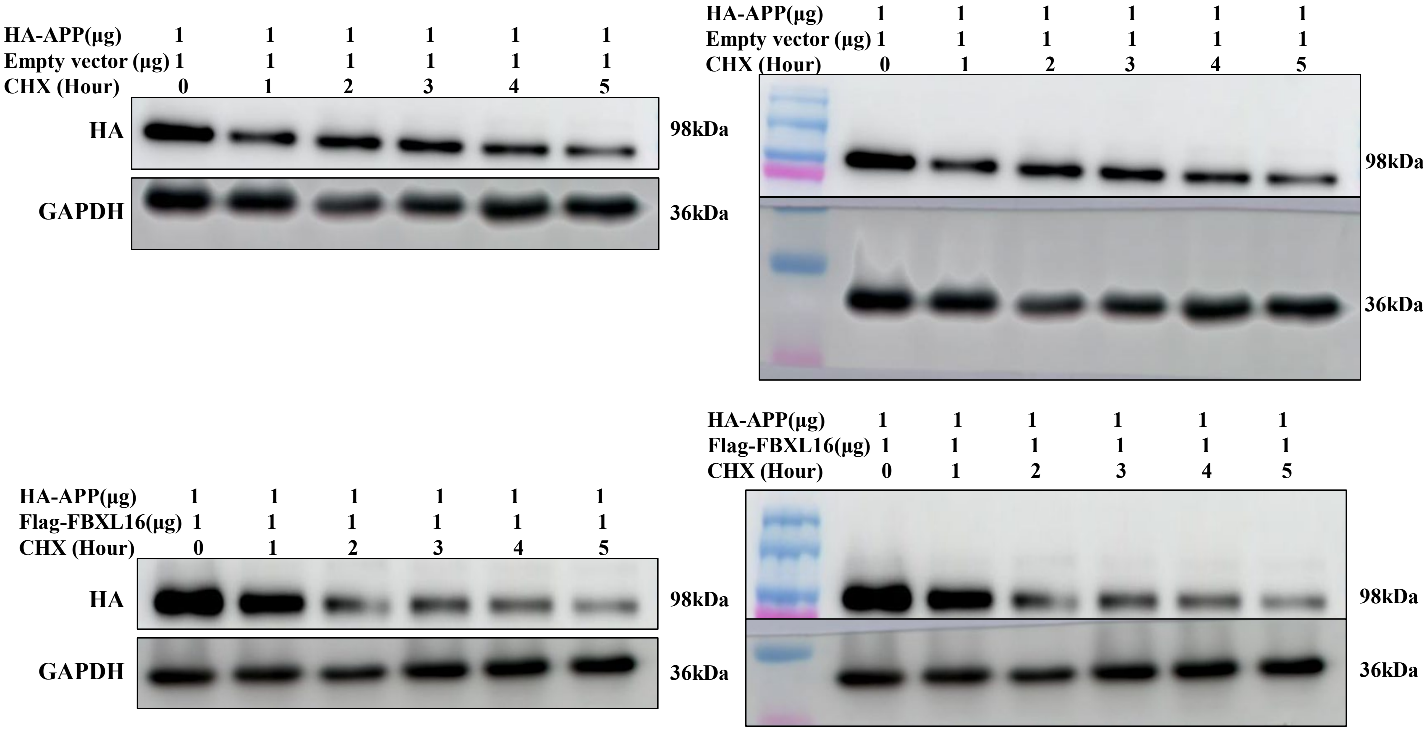

B

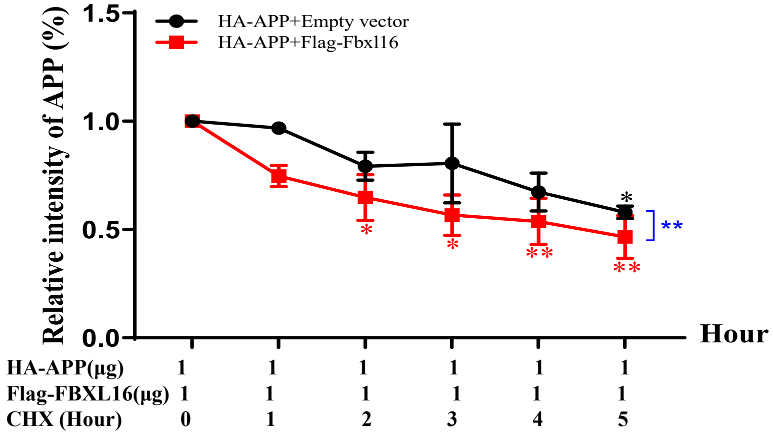

Supplementary Figure 6

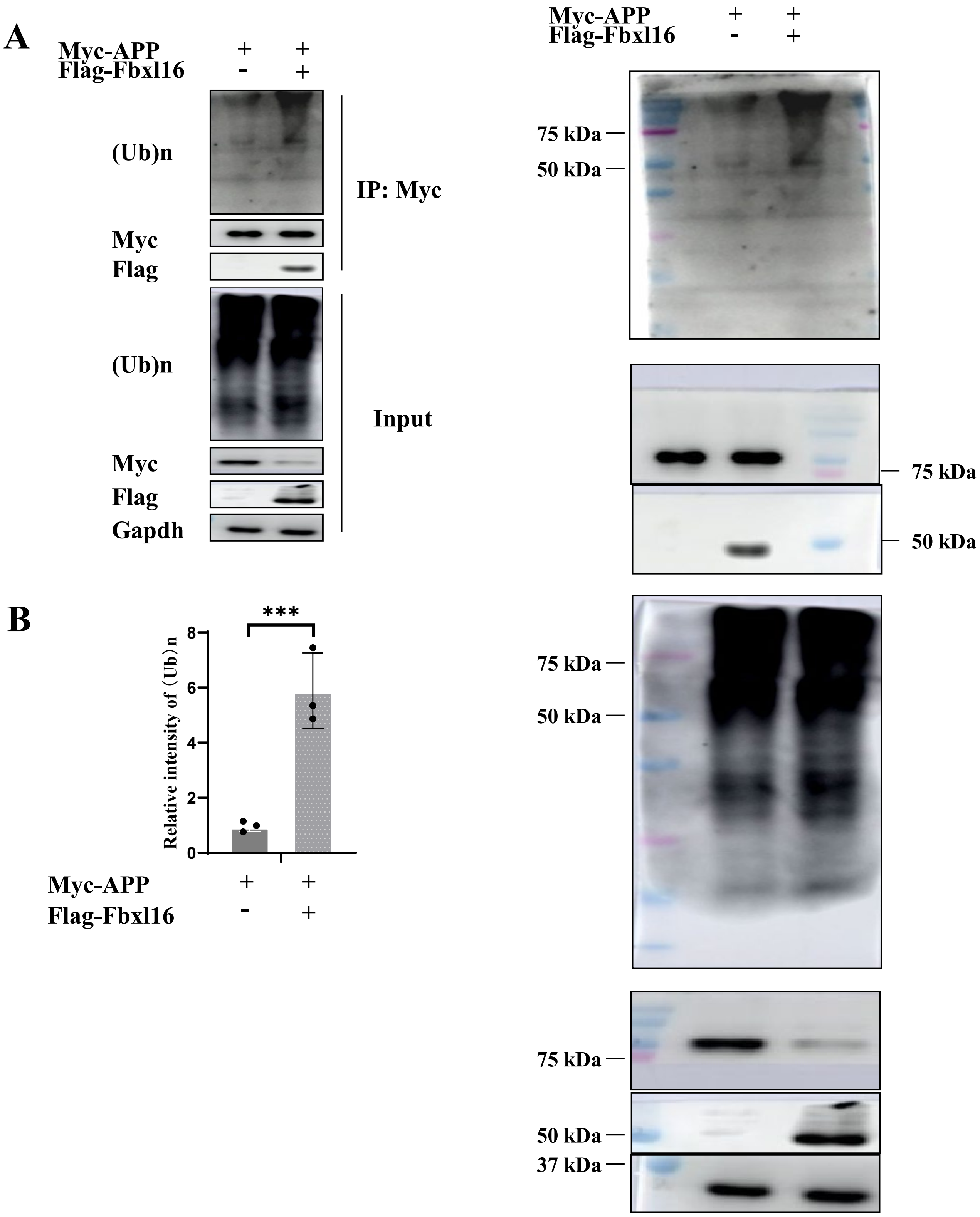

Supplementary Figure 7

A

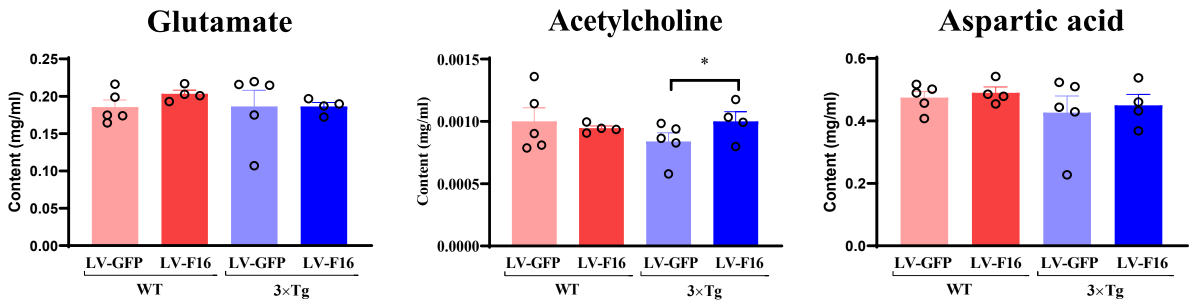

B

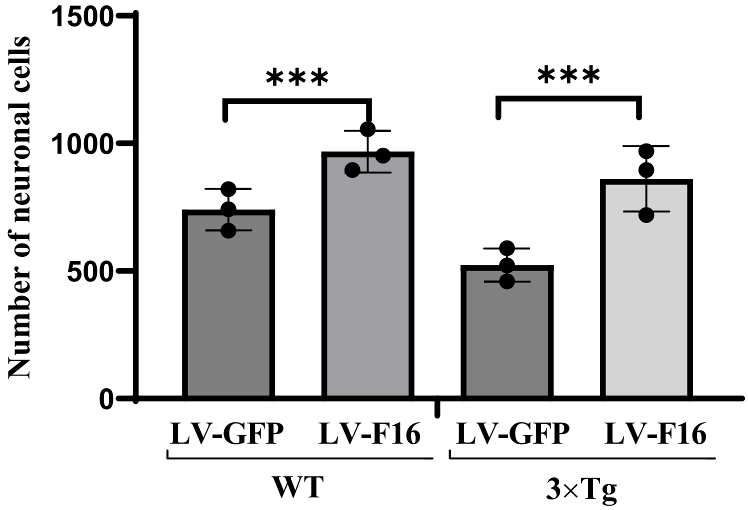

Supplement: Supplementary file 1 — Supplementary Material 1 [file 40364_2024_691_MOESM1_ESM.pdf]
